# Are There Consistent Grazing Indicators in Drylands? Testing Plant Functional Types of Various Complexity in South Africa’s Grassland and Savanna Biomes

**DOI:** 10.1371/journal.pone.0104672

**Published:** 2014-08-11

**Authors:** Anja Linstädter, Jürgen Schellberg, Katharina Brüser, Cristian A. Moreno García, Roelof J. Oomen, Chris C. du Preez, Jan C. Ruppert, Frank Ewert

**Affiliations:** 1 Range Ecology and Range Management Group, Botanical Institute, University of Cologne, Cologne, Germany; 2 Institute of Crop Science and Resource Conservation, University of Bonn, Bonn, Germany; 3 Department of Soil, Crop and Climate Sciences, University of the Free State, Bloemfontein, Republic of South Africa; Dauphin Island Sea Lab, United States of America

## Abstract

Despite our growing knowledge on plants’ functional responses to grazing, there is no consensus if an optimum level of functional aggregation exists for detecting grazing effects in drylands. With a comparative approach we searched for plant functional types (PFTs) with a consistent response to grazing across two areas differing in climatic aridity, situated in South Africa’s grassland and savanna biomes. We aggregated herbaceous species into PFTs, using hierarchical combinations of traits (from single- to three-trait PFTs). Traits relate to life history, growth form and leaf width. We first confirmed that soil and grazing gradients were largely independent from each other, and then searched in each biome for PFTs with a sensitive response to grazing, avoiding confounding with soil conditions. We found no response consistency, but biome-specific optimum aggregation levels. Three-trait PFTs (e.g. broad-leaved perennial grasses) and two-trait PFTs (e.g. perennial grasses) performed best as indicators of grazing effects in the semi-arid grassland and in the arid savanna biome, respectively. Some PFTs increased with grazing pressure in the grassland, but decreased in the savanna. We applied biome-specific grazing indicators to evaluate if differences in grazing management related to land tenure (communal versus freehold) had effects on vegetation. Tenure effects were small, which we mainly attributed to large variability in grazing pressure across farms. We conclude that the striking lack of generalizable PFT responses to grazing is due to a convergence of aridity and grazing effects, and unlikely to be overcome by more refined classification approaches. Hence, PFTs with an opposite response to grazing in the two biomes rather have a unimodal response along a gradient of additive forces of aridity and grazing. The study advocates for hierarchical trait combinations to identify localized indicator sets for grazing effects. Its methodological approach may also be useful for identifying ecological indicators in other ecosystems.

## Introduction

Resource availability and disturbances are widely recognized as key drivers of plant community structure and composition [1]. Rangeland vegetation is shaped by recent grazing pressure, together with multiple combinations of land use history and available resources [2],[3]. In the case of dryland rangelands, management effects are particularly difficult to detect against the background of a high environmental variability [4], [5].

There is a growing need for ecological indicators that provide aggregated information for assessing states and trends of vegetation dynamics [6]. For this purpose, it is crucial to decide on an optimum type and level of aggregation of vegetation characteristics [7]. Plant functional types (PFTs) are a typical example for an aggregation of plant species, and PFTs are frequently applied as indicators for the state of dryland rangelands [8]. PFT classification is a widely supported method in data analysis to aggregate species and to reveal a consistent response of ecosystems, irrespective of species identities [9]. The underlying rationale is that different species within a PFT share traits that show a similar response to grazing disturbance [10]. Hence, PFTs can be powerful indicators for rangeland condition due to their functional relation to ecosystem processes [11].

A-priori classifications of PFTs have been criticised for neglecting the specific environmental settings and the evolutionary history of the study area [12]. However, individual plant traits are typically highly interrelated, and thus pairs or groups of traits usually co-vary [13]. This complexity of relations among traits can usually and effectively be summarized by aggregating individual traits to one or few key traits that capture a large proportion of variation in vegetation responses to grazing [14]. Life forms [15] and growth forms are early and well-known expression of such correlation among traits [13],[16]. They are extensively used to describe functional plant responses to grazing [17], [18].

However, despite the vast literature on plant trait and PFT responses to grazing in drylands, there is still no consensus if an optimum level of trait aggregation exists for detecting grazing effects in these ecosystems [14], [19]. This is particularly true for transferring grazing responses to sites with different climatic and/or edaphic aridity, and for upscaling from the plant community to the biome level and beyond. To address plant responses to grazing, Díaz et al. [20] propose life form, life history (annual versus perennial), plant height and architecture as a good trait set to define PFTs. As a minimum set for herbaceous plants they suggest life history and height or habit. For the more elaborated climate-grazing category of ‘dryland environments with a long evolutionary history of grazing’ (such as Africa’s drylands), they recommend for herbaceous plants to distinguish between short and tall graminoid growth forms of C_3_ or C_4_ metabolism, and between forbs of prostrate, erect or tall architecture. However, these recommendations of Díaz et al. [20] are sometimes contradictive as, for example, life history is included in the minimum trait set, but not in some of the more elaborated climate-grazing categories. Hence, even these general recommendations are not consistent with respect to which level of trait aggregation (or trait sets) should be used in drylands.

In the past decades, dryland rangelands in the Republic of South Africa (RSA) have undergone profound changes in grazing management, which in turn triggered changes in plant community composition [21]. Grazing management itself is substantially influenced by the form of land ownership (further referred to as tenure system). Most rangelands are either free-hold, or communal rangelands [21]. Free-hold, commercial farms (about 70% of all land used in the RSA) are generally considered to be in good condition, and, are typically managed using a rotational grazing system at moderate stocking densities [22]. In contrast, communal rangeland (about 14% of South Africa’s used land) holds about half of all livestock in RSA and is often associated with land degradation as a result of continuous grazing at high stocking densities. However, the relative extent of rangeland degradation under freehold compared to communal tenure has been increasingly debated in the past years [23].

In this context, surprisingly little is known about differential tenure effects along climatic aridity gradients, or across different biomes. Two of the major rangeland biomes in southern Africa are grasslands and savannas [24], [25]. The vast majority of rangeland studies has focused on single biomes, most often on the grassland biome [26]–[28] followed by the savanna biome [29]–[31]. A recent study analysed impacts of heavy grazing on plant species richness across six South African rangeland biomes [21], but did not differentiate between land tenure systems. Literature on global, cross-biome comparisons of vegetation responses to grazing and variable climate is also increasingly available [4], whereas investigations on tenure-related management effects across biomes are still scarce. In particular, cross-biome comparisons have rarely been done with respect to the functional response of plant aggregations to grazing at local and tenure system level, and with respect to possible confounding effects of abiotic site conditions.

Accordingly, the objectives of this study were two-fold. First, we aimed to identify functional plant aggregations which were good and consistent grazing indicators across South Africa’s grassland and savanna biomes. For this purpose, we developed a novel, standardized approach to quantify how trait-based plant aggregations of various complexity responded to grazing and to other environmental conditions. Second, we aimed to assess tenure-related effects of grazing management on rangeland vegetation with the aid of these indicators. Even though controlled experiments usually render the most definitive results as variables except the treatment are held constant, they are not feasible for addressing ecological questions at the scale of ecosystems and landscapes [32]. Hence, we used a comparative field study, which made use of the prevailing (semi-) natural conditions and their management-induced variation.

In detail, we addressed the following research questions:

1. Are soil and grazing gradients independent from each other, making an identification of grazing indicators principally possible?
2. Is there an optimum hierarchical level of functional aggregation for assessing grazing effects on rangeland vegetation, shown by a consistent response of PFTs to grazing across biomes?
3. Can we detect differences in vegetation state between tenure systems in the two biomes, and can these differences be clearly related to grazing effects?

We hypothesized that (1) soil and grazing gradients are largely independent from each other, that (2) an optimum level of trait aggregation exists, and that (3) communal farms display stronger grazing-induced vegetation changes.

## Materials and Methods

### Study Areas

**Figure 1:**
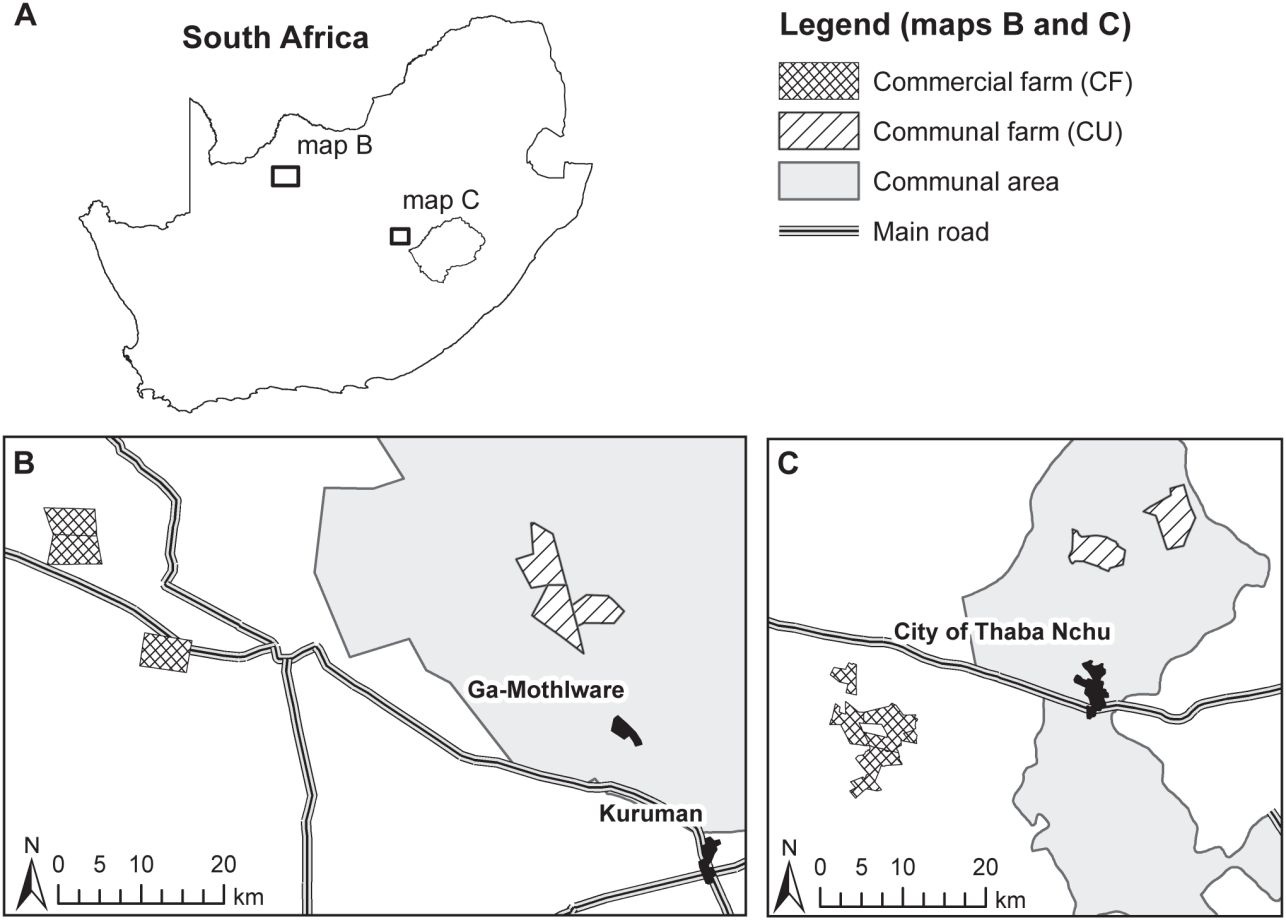
Study areas and farms in South Africa’s grassland and savanna biomes. The large map (A) indicates the position of the two study areas in South Africa. The detailed maps give the position of commercial and communal farms in the savanna biome (B) and in the grassland (C). Communal areas are situated in the former homeland Bophuthatswana.

Study sites were located in South Africa’s Free State province (grassland biome), and in the Northern Cape (savanna biome; Figure 1). The two areas greatly differ with respect to climatic aridity, soil conditions and species pools (for details see Table 1). Most importantly, the savanna site has an arid climate (mean annual precipitation 417 mm), while the climate of the grassland site is semi-arid (mean annual precipitation 572 mm). However, the grass layer is in both cases dominated by perennial C4 tuft grasses [33], [34]. The land use history of both study areas is representative for South Africa’s grassland [35] and savanna biome [36]. In the savanna biome, bush encroachment was already observed in the study region in the 1870s and is ongoing until today.

**Table 1:** Biome and tenure system characteristics, and soil differences between piosphere and pasture plots of the tenure systems in the two biomes.

Biome	Grassland				Savanna			
Temperature [°C]								
Max & min (January/July)	31/15 (maximal), 16/−2 (minimal)				32/17 (maximal), 18/1 (minimal)			
Annual precipitation								
MAP [mm]/CV [%]	572/30				417/42			
Rainy season	Summer (October to April)				Summer (September to April)			
Dominant soil type	Lixisols (deep soils with clay-enriched subsoil; on sediments or shale)				Arenosols (deep aeolian sands, underlain by calcrete)			
Vegetation type	Moist Cool Highveld Grassland (eutrophic grassland)				Kalahari Plains Thorn Bushveld			
Dominant plant species	Perennial C_4_ tuft grasses (*Themeda triandra* dominant in natural vegetation; *Triraphis andropogonoides, Eragrostis superba*)				Grass layer: Perennial C_4_ tuft grasses (e.g. *Stipagrostis uniplumis*), tree layer: *Acacia erioloba, Boscia albitrunca*			
Rangeland management								
Recommended stocking density [ha LSU^−1^]	5–10				9–16			
Tenure system	Commercial (CF)		Communal (CU)		Commercial (CF)		Communal (CU)	
Herd composition	Cattle, few sheep		Cattle, sheep		Cattle		Cattle, goats, donkeys	
Herd mobility	Rotation between camps, with long rest of winter camps		Continuous, with daily movements from settlement		Permanent rotation between camps		Continuous, with daily movements from settlement	
Stocking density [ha LSU^−1^]	5.5±2.4		5.2±1.2		14.2±7.1		10.9±3.7	
Edaphic conditions	Piospheres	Pastures	Piospheres	Pastures	Piospheres	Pastures	Piospheres	Pastures
Sand content [%]	43.3±9.6 ab	47.8±10.3 a	38.2±7.9 b	45.2±12.2 ab	94.1±1.3 ab	94.9±2.7 a	94.0±1.9 ab	92.9±2.1 b
Silt content [%]	28.2±5.7	25.4±6.9	26.1±2.2	25.1±7.7	2.6±1.6	2.0±1.4	2.6±0.8	3.0±1.6
Clay content [%]	27.7±8.2 ab	25.7±7.7 a	34.4±7.9 b	29.2±14.2 ab	3.1±1.6 a	3.5±1.9 a	4.1±1.1 ac	4.5±0.8 bc
Total nitrogen [g kg^−1^]	1.23±0.34 a	1.10±0.23 a	1.10±0.22 a	1.01±0.20 b	0.16±0.07 ab	0.12±0.04 a	0.19±0.07 b	0.18±0.05 b
Available phosphorus [mg kg^−1^]	7.1±6.8	5.3±4.4	4.1±2.1	5.2±3.5	4.7±5.1 a	1.9±0.9 b	2.9±2.1 b	2.5±1.4 b

Climate data: Bloemfontein (29.10°S, 26.30°E), ca. 30–70 km distance to grassland sites; Kuruman (27.43°S, 23.45°E), ca. 35–75 km distance to savanna sites. MAP: mean annual precipitation of hydrological years (July–June); CV: coefficient of variation for MAP. For both meteorological stations, only years without data gaps were used for calculations; data source: http://climexp.knmi.nl. Bloemfontein data 1904–2011 (n = 100), Kuruman data 1905–1997 (n = 62). Soil types are given as WRB type [74]; grassland soils after [50]; savanna soils after [24]. Vegetation characteristics of the grassland after [25]; savanna: after [24]. Herd composition, mobility and farm-specific stocking densities for 2011 were derived from pers. comm. with farmers and provided by C. Naumann. Please note for stocking density that a larger number reflects a lower density. Edaphic conditions are given for the topsoil (0–20 cm) of vegetation plots; letters indicate significant differences within a biome (Tukey’s HSD, *p*<0.05; standardized data).

### Study Set-Up and Sampling Design

To address our research questions, we used a cross-biome comparison with a unified sampling design. Specifically, we sampled livestock grazing gradients in two major South African biomes (mainly characterized by different climatic aridity), and further stratified for grazing management. For this purpose, two tenure systems were selected in both biomes, farms with commercial production under freehold tenure (commercial farms, CF), and farms under communal tenure (CU). Tenure systems differ with respect to land use history, ownership, access regime, and herd management such as the timing and frequency of herd movements (Table 1; for details on land use history see Text S1). With our sampling design we aimed to maximize detectable effects of grazing, and to minimize potentially confounding effects of other environmental conditions. As in other comparative studies of real-world ecosystems (e.g., [18], [21]), we were not able to completely control for the high environmental variability typical for drylands. By sampling unburnt sites with similar topography, lithology and soil type, we made an effort to control for as many environmental factors as possible (see Text S1 for details).

### Ethics Statement

No specific permissions were required for research locations and activities, as no endangered or protected species were involved, and as field work was carried out on unprotected private or communal land. The research area in the grassland biome encompassed a rectangle between 28.95°S, 26.46°E and 29.41°S, 27.00°E; that in the savanna biome a rectangle between 26.98°S, 22.65°E and 27.56°S, 23.47°E within which all selected farms and communal areas were located (Figure 1). In both research areas, we obtained permissions from the local agricultural offices; in the grassland biome from the Thaba Nchu Agricultural Office of the Free State Department of Agriculture and Rural Development (10 Riet Street, 9301 Thaba Nchu, Free State), and in the savanna biome from the Mothibistad Agricultural Office of the Northern Cape Department of Agriculture, Land Reform and Rural Development (PO Box 26, 8460 Kuruman, Northern Cape). We additionally obtained private permissions from all freehold farmers to work on their land, as well as permissions from all headmen and local municipalities to work in communal areas (grassland biome: Mangaung Local Municipality, P.O. Box 3704, 9300 Bloemfontein, Free State; savanna biome: Ga-Segonyana Local Municipality, Private Bag X1522, 8460 Kuruman, Northern Cape). Data underlying this study are made publicly available on the homepage of the Range Ecology and Range Management Group at the Botanical Institute, University of Cologne, Germany (http://www.botanik.uni-koeln.de/range_ecology.html).

### Sampling

Field work was conducted in the growing season 2010/11. Rainfall in the related hydrological year (July 2010–June 2011) was exceptionally high in the grassland, i.e. 952 mm (66 % above long-term mean) and high in the savanna (462 mm, +11 %). In the grassland, previous rainfall (2009/10) was also high. In the savanna, average stocking densities in 2011 on CF and CU farms were ca. 2–3 times lower than in the grassland (Table 1; please note that a larger number stands for a lower density). This reflects the lower primary and secondary productivity in the more arid savanna environment. Accordingly, stocking densities in both biomes fell within the range of recommended numbers, but were (as expected) higher in communal areas. For each farm or community, a representative paddock (section of the rangeland on CU farms) was selected, including one permanent, artificial water point (>50 years). Following recommendations of Shipley [37], our sampling approach was designed to best measure the environmental gradient of interest (here the gradient of grazing pressure). This was done (i) by choosing sites that maximized the range of grazing pressure; and (ii) by concentrating our sampling effort on points where grazing pressure is known to be changing most quickly. For this purpose, we combined transect-based and random sampling. In the small piosphere around the water point where grazing pressure is known to change considerably over a short distance [28], [38], a transect was sampled from the water point outward until average pasture conditions were reached, based on a visual assessment of the physical evidence of grazing. Transect length varied between 48 and 186 m in grassland and between 180 and 738 m in the savanna. Evenly spaced along each transect, six vegetation plots of 5×5 m in the grassland, and 10×10 m in the savanna were laid out, with the first five plots (‘piosphere plots’) in the piosphere, and the sixth plot outside (‘pasture plot’). We randomly established 9–13 additional pasture plots on each pasture, limiting sampling to the habitat type of plains. Minimum distance between plots exceeded twice their side length. Plant species abundance was assessed on plots by visual estimates of percentage ground cover. Estimates were done independently by two to three experienced observers. In total, 213 plots were sampled, 35 and 30 in the piosphere and 86 and 62 pasture plots on grassland and in the savanna, respectively. These plots were distributed across 5 CF versus 2 CU on grassland and 3 CF versus 3 CU in the savanna.

#### Soil variables

The proportion of the soil surface covered with coarse material, i.e. particles >2 mm (Skeleton), was visually estimated. We collected five topsoil samples per plot (0–20 cm), and mixed them for a bulk sample. Soil analyses were carried out on soil fractions <2 mm using standard methods [39]. Particle size was determined by sieve-pipette method. Soil pH was determined in a 1∶2.5 water suspension. Plant-available phosphorus (P_av_) was measured as extractable (Olsen) P (1 mol dm^−3^ NaHCO_3_ at pH 8.5) and determined colorimetrically [40]. Exchangeable Ca^2+^, K^+^, Mg^+^ and Na^+^, as well as Cation Exchange Capacity (CEC) were determined by atomic absorption (1 mol dm^−3^ NH_4_OAc at pH 7). Extractable micronutrients (Fe^2+^, Mn^2+^, Cu^2+^ and Zn^2+^) were determined with the DTPA method [41]. Total carbon (C_tot_) and total nitrogen (N_tot_) were analysed by dry combustion with a CN analyser (NA 1500). As no inorganic C was detectable, total C was regarded as organic C.

#### Variables reflecting grazing pressure

Grazing impact on plots through defoliation and trampling was assessed (i) via a plot’s distance [m] from the water point, which was expressed as raw (untransformed) distance. Furthermore, we visually estimated the ground cover of (ii) moribund biomass (previous year’s standing dead), (iii) plant litter) and (iv) bare soil, which is the area of soil not covered by vegetation, litter or skeleton [42]. The increasing distance from water points is frequently used as a proxy for decreasing grazing pressure [12], [28]. A high proportion of bare soil and a low cover of litter and moribund biomass indicate high grazing pressure [42], [43]. Further (v), we combined physical evidence of grazing (trampling, dung, and the removal of standing biomass) in an expert assessment of recent grazing pressure. Plots scored from 1 (none) to 9 (extreme grazing pressure). Little (3), moderate (5) and heavy (7) grazing pressure corresponded to an estimated removal of standing biomass of 20, 40 and 60%, respectively [see 44]. Finally (vi), the ground cover of woody plants was estimated in the savanna to capture the extent of bush encroachment. Bush encroachment was on several farms clearly discernable in the field, particularly from the age structure and density of *Acacia mellifera* populations, and was included in our study because it is often the consequence of grazing mismanagement [45].

### Data Analysis

We developed a novel approach to quantify PFTs’ responses to grazing pressure, which combines a hierarchical definition of trait-based PFTs with multivariate statistics and model selection procedures. In the first step (related to question 1), we tested if local soil and grazing gradients were largely independent from each other, using data from all plots. In the second step (addressing question 2), we aggregated the community composition into relative abundances of plant functional types (PFTs) per plot. PFTs were defined via three hierarchical levels of trait combinations, from single- to three-trait PFTs. To evaluate species turnover within plant communities and to compare PFT responses to species responses, we also aggregated local community composition into ordination scores. We then applied two criteria to identify the indicative potential of PFTs in a certain biome; i.e. if they exhibited a strong response to grazing pressure (sensitivity), and if they were little confounded by other ecosystem properties (specificity). We used data from all plots to capture the full range of grazing pressure. In a third step (related to question 3), we used PFTs that were identified in the previous step as good grazing indicators to assess tenure effects on rangeland vegetation, comparing effects on piosphere and pasture plots.

### Aggregation of Environmental Variables

To aggregate environmental parameters into composite variables and to evaluate correlation between grazing and soil gradients, we performed PCA with varimax rotation. As precision and units of data varied, we used the correlation matrix of the raw data [46]. We combined the six parameters reflecting grazing pressure with 17 soil variables (Table S1) and performed PCA separately for each biome. Scree-plots were used to assess the appropriate number of principal components (PC) with eigenvalues >1. In each biome, we used a PC with high factor loadings of parameters reflecting grazing pressure (‘grazing-related PC’) as a predictor of grazing pressure in subsequent analyses.

### Aggregation Procedures

#### Community composition

We aggregated plant community composition via two alternative ordination procedures: DCA (CANOCO version 4.5 [47]) and NMDS (PC-ORD version 5 [48]). Analyses were performed with log-transformed species’ cover values. In the savanna, we lumped unidentified taxonomic groups as ‘annual forbs’ and ‘perennial forbs’. Relative cover of those groups was always <5 % (median values 0.1 % and 2.0 %). For DCA, we used the default settings of CANOCO, but a down-weighting of rare species was performed. Despite some criticism with respect to the underlying distance model and for the approach of detrending, DCA is a standard method for unconstrained ordination which provides a meaningful scaling of the ordination space [49]. Note that we refrained from interpreting the second DCA axis due to concerns about interpretability [49]. For NMDS, we chose Bray–Curtis dissimilarity as a distance measure [50]; see Text S1 for details.

#### Plant functional types

We used a hierarchical, trait-based approach to aggregate species into PFTs. It roughly followed the methodology proposed by McIntyre et al. [16] for defining PFTs which are responsive to disturbance. McIntyre et al. [16] identified different sets of traits as being relevant for each ‘major life-form group’, and proposed to apply a higher grain of classification for life-form groups which are ‘locally relevant’ (i.e. occurring). Following these recommendations, hierarchical levels in our study were based on the following traits (see Table 2): (i) For single-trait PFTs, we only considered dormant bud position (life form; [15]); (ii) for two-trait PFTs, we additionally considered graminoid or non-graminoid growth form; (iii) for three-trait PFTs, we additionally considered leaf width, distinguishing narrow-leaved (<5 mm), broad-leaved (5–10 mm) and very broad-leaved (>10 mm). This final classification was only performed for annual and perennial graminoids (hereafter grasses). The main reason for this was that species from these life-form groups were in both study areas dominant, and thus ‘locally relevant’ [16]. Functional relevance of leaf width was another reason: First, leaf width of grasses (but not of forbs or shrubs) could be used as a proxy for specific leaf area, which has a high functional relevance for plants’ resource acquisition and conservation strategies [38], [51]. Second – in contrast to grasses – forb leaf width is not reported to have functional relevance for disturbance response [16]. To obtain required trait data, we checked taxonomic and ecological literature (e.g., [52]–[54]); see Table S4 for species’ trait values.

**Table 2:** Hierarchical, three-level approach for the definition of plant functional types (PFTs) based on categorical functional traits.

Aggregation leveland considered traits	Trait categories and resulting plant functional types											
Single-trait PFTs(trait: dormant budposition = Raunkiær’slife form)	Categories	Buds belowground		Buds on persistentshoots, <50 cmto ground	Buds at or near ground				Buds within seeds			
	% of taxa^a^	8/2		11/10	55/>46				26/>42			
	PFT acronym^b^	(Ge)		Ch	H				T			
	PFT name^c^	Geophytes		Chamaephytes( = shrubs)	Hemicryptophytes( = perennial forbs & grasses)				Therophytes ( = annual plants)			
Two-trait PFTs(added trait: growthform; for herbaceousPFTs only)	Categories	Forbs	Grasses		Forbs	Grasses			Forbs	Grasses		
	% of taxa	6/2	2/0		29/>30	26/29			20/>32	5/9		
	PFT acronym	(GeF)	(GeG)		HF	HG			TF	TG		
	PFT name				Perennial forbs	Perennial grasses			Annual forbs	Annual grasses		
Three-trait PFTs(added trait: leafwidth; for grassPFTs only)	Categories					Linear	Lanceolate	Ovate		Linear	Lanceolate	Ovate
	% of taxa					11/14	13/15	2/0		1/2	4/7	0/0
	PFT acronym					HG lin	HG lan	HG ov		(TG lin)	(TG lan)	(TG ov)
	PFT name					Narrow-leavedperennial grasses	Broad-leavedperennial grasses	Very broad-leavedperennial grasses				

a The percentage of plant species belonging to a certain PFT is given separately for the grassland/for the savanna biome.

b PFT acronyms describe the hierarchical combination of traits. For single-trait PFTs, acronyms are based on the first 1–2 letters of Raunkiær’s life form classification [15]; for two-trait PFTs, acronyms for growth form are added (G = grasses, F = forbs); for three-trait PFTs, acronyms for leaf width are added (lin = linear (narrow-leaved), <5 mm; lan = lanceolate (broad-leaved), 5–10 mm, ov = ovate (very broad-leaved), >10 mm). PFTs which acronyms are in brackets were not included in further analyses due to their low frequency and low relative abundance on plots.

c For two- and three-trait PFTs, names are only given if PFTs were retained in subsequent analyses, at least in one biome.

In contrast to the original hierarchical approach by McIntyre et al. [16], we considered trait combinations from all three hierarchical levels. This rendered 16 potential PFTs (Table 2). Six PFTs were not used in further analyses due to their low frequencies and relative abundances (Table 2, PFT acronyms in brackets). Another two PFTs (Ch: chamaephytes (shrubs) and HG ov: very broad-leaved perennial grasses) were for the same reason excluded from the savanna dataset. This left eight PFTs for the savanna and ten (i.e. two additional ones) for the grassland. Among the eight PFTs which were shared between biomes, two were single-trait PFTs (H: hemicryptophytes (perennial grasses and forbs), and T: therophytes (annuals)); four were two-trait PFTs corresponding to ‘major life-form groups’ defined by McIntyre et al. ([16]; see above), and two were three-trait PFTs (HG lin: narrow-leaved perennial grasses, HG lan: broad-leaved perennial grasses).

### Testing the Response of PFTs to Grazing and Soil Conditions

To identify plant aggregations which were good ecological indicators for grazing pressure in the two biomes, we used linear models (LM) with land tenure as categorical and PCA-derived variables (reflecting grazing pressure and abiotic site conditions) as linear predictors. Response variables were the relative abundances of PFTs. For comparison reasons, we also constructed linear models for the scores of the two NMDS axes, and of the first DCA axis (reflecting species turnover within plant communities). To achieve homoscedasticity and normality, PFT data were arcsine-transformed. Separate models were calculated for each response variable in each biome. Model simplification was done via a likelihood ratio test (Type 1 test) of all possible subsets of effects. We only retained variables that significantly contributed to model performance at *p*<0.05.

We cross-checked the results by performing an alternative best-subset selection based on the Akaike Information Criterion (AIC). We calculated Delta AIC (ΔAIC) values for the two alternative final models, which measures the performance of the best model (based on the likelihood ratio test) relative to the best AIC-based final model [55]. We quantified the strength of response to grazing and to abiotic site conditions via effect sizes (classical Eta-squared, η^2^). In each biome, we identified good grazing indicators via two criteria, (i) a sensitive response to grazing, shown by a significant contribution of the grazing-related PC to the final LM, and (ii) a specific response (little confounded by variation in other ecosystem properties), shown by the largest effects size of the grazing-related PC in the final LM. Indicators’ response consistency across biomes was evaluated by comparing their sensitivity, specificity, and direction of response.

### Assessing Land Tenure Effects using PFTs responsive to Grazing

In a final step, we evaluated tenure-related management effects on rangeland vegetation with the aid of PFTs identified as good grazing indicators. We used a partially nested, mixed-model ANOVA design, and included ‘farm’ (nested in tenure and biome) as a random factor, and the fixed factors ‘tenure’ (CF versus CU), ‘biome’ (grassland versus savanna) and ‘plot type’ (piosphere versus pasture plots). Response variables were arcsine-transformed relative abundances of PFTs with a specific response to grazing at least in one biome. Homogeneous groups of factor combinations were identified by post-hoc comparisons (Tukey’s HSD; *p*<0.05).

## Results

### Testing Independence of Soil Gradients and Grazing Gradients (Question 1)

PCA revealed five main environmental gradients in both biomes (Table S1). Subsequent axes explained <36% of total variation in the grassland dataset and <35% in the savanna, and were not considered further. In the grassland, grazing was reflected by PC 3, which explained only 10% of total variation within the data set. The grazing gradient reached from plots with little grazing impact at greater distances to water points, and with high cover values for litter and moribund material, to plots at close distances to water points with a high proportion of bare soil and visible signs of severe grazing. PC 1, 2 and 4 reflected gradients in edaphic conditions, with PC 1 describing a gradient between plots with high clay content and high CEC, and those with high sand content. PC 2 described a gradient in mineral nutrients.

In the savanna, grazing was reflected by PC 2 which explained 18% of variation among plots, i.e. considerably more than in the grassland. PC 1 was mainly associated with changes in bush cover, soil organic matter and nitrogen. Gradients in edaphic conditions (particularly soil texture) were bundled by PC 3–5. While we found a weak correlation between management and soil parameters in the grassland, grazing and three mineral nutrients (P, K and Zn) were correlated in the savanna (factor loadings >4 on same PC; Table S1).

### Response Consistency across Biomes (Question 2)

#### Response of community composition

**Figure 2:**
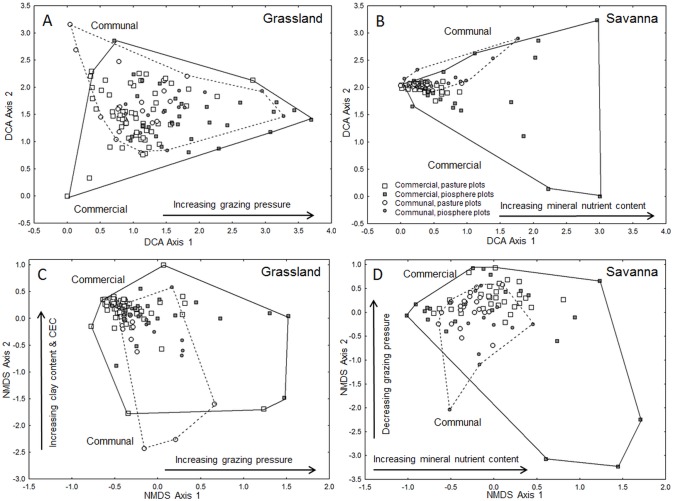
Ordination diagrams of herbaceous community composition. Ordinations are based on two alternative procedures (A, B: detrended correspondence analysis, DCA; and C, D: non-metric multidimensional scaling, NMDS). They visualize differences between piosphere plots and pasture plots on commercial farms and communal farms in South Africa’s grassland biome (A, C) and savanna biome (B, D). Close plots feature a similar species composition, remote plots are more dissimilar. Interpretation of ordination axes follows final linear models with PCA-derived composite variables as predictors. In the grassland biome, a gradient of increasing grazing pressure underlies species turnover along the first ordination axes; in the savanna, it is a gradient of mineral nutrient content in the topsoil (0–20 cm). Note that we refrained from interpreting the second DCA axes due to concerns about their interpretability.

In both biomes, piosphere and pasture plots from the two tenure systems were not clearly separated in the ordination spaces (Figure 2). Both ordination methods rendered essentially similar results. They agreed for the savanna that, along the main ordination axes (DCA 1 and NMDS 1), variation among piosphere plots was higher than that among pasture plots (Figure 2B and 2D). In congruence to our expectations, this reflected steeper environmental gradients close to water points. However, our expectations were not met for the grassland, where variation among pasture plots was also high (Figure 2A and 2C).

**Figure 3:**
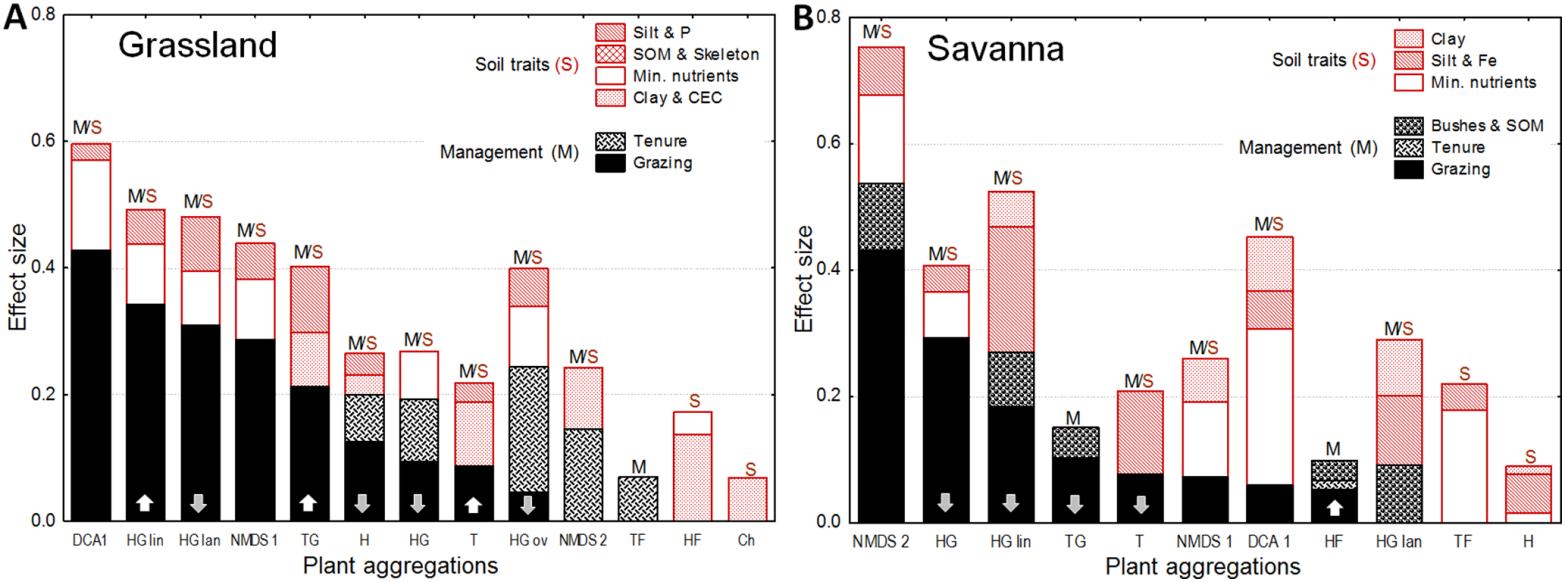
Response of plant aggregations to management and soil conditions in the grassland (A) and in the savanna biome (B). For each plant aggregation, bars denote the proportion of explained variance (given as effect sizes, η^2^) in best-fitting linear models, associated with biome-specific principal components and land tenure. Parameters are ordered by their effect sizes, starting with the grazing-related principal component. Arrows facing upwards indicate a positive response to increased grazing, and arrows facing downwards indicate a negative response. Note that negative or positive responses to grazing cannot be assigned to ordination axes. DCA 1 = plot scores on first DCA axis. For abbreviations of PFTs, refer to Table 2.

In the grassland biome, the grazing-related PC explained the highest proportion of species turnover along the first axis of the two ordination procedures (DCA 1: 43%, NMDS 1: 29%; see Figure 3), followed by a variable group reflecting mineral nutrients (PC 2) and a PC reflecting changes in silt and P content of the topsoil (PC 5; see Tables S2 and S3 for details of final linear models). Unexpectedly, the most important predictor for species turnover in the savanna was not grazing pressure (PC 2; explained variance only 6%) but a gradient in mineral nutrients (PC 3; explained variance 25%). Other soil parameters (PC 5 related to topsoil clay, and PC 4 to silt and Fe content) were of minor importance. In both biomes, community composition in the savanna (DCA 1) was not explained by differences in land tenure. To substantiate our claim that certain ordination axes reflected a grazing gradient, we performed correlations between these axes and reciprocal distances to the water point, as this estimate reflects grazing intensity better than distance [56]. In the grassland, both DCA 1 and NMDS 1 showed a strong positive correlation to reciprocal distance (*p*<0.001). Coefficients of determination (*r^2^*) indicated that reciprocal distance explained 23.4 % of variation in DCA 1 and 21.9 % of variation in NMDS 1. In the savanna, a strong negative correlation was found between NMDS 2 and reciprocal distance (explained variance 53.6 %; *p*<0.001).

In the grassland biome, the response of narrow-leaved and broad-leaved perennial grasses (HG lin and HG lan) to grazing and soil conditions resembled the main changes in community composition (DCA 1 and NMDS 1; see Figure 3). In the savanna, perennial grasses’ responses resembled the NMDS 1 responses to environmental conditions, but none of the tested PFTs resembled DCA 1 responses.

#### Response consistency across biomes – sensitivity, specificity and direction of response

Great differences occurred with respect to PFTs’ sensitivity and specificity across biomes (Table 3 and Figure 3). Overall, 70% of PFTs in the grassland biome (savanna: 63%) showed a sensitive response to grazing pressure, and 50% (savanna: 38%) were also specific in their response, meaning that they did not respond more strongly to other environmental conditions. Six from ten PFTs had a specific response to grazing at least in one biome, but only one (annual grasses) in both. Moreover, half of the six PFTs with a specific response in one biome were insensitive to grazing effects in the other (Figure 3).

**Table 3:** Response consistency of six PFTs which are good (specific) indicators for grazing pressure at least in one biome.

PFT		Response to grazing^a^		Response consistency		
Description	Acronym	Grassland	Savanna	Sensitivity	Specificity	Direction
Annual grasses	TG	specific ↑	specific ↓	yes	yes	-
Perennial grasses	HG	sensitive ↓	specific ↓	yes	-	yes
Small-leaved perennial grasses	HG lin	specific ↑	sensitive ↓	yes	-	-
Broad-leaved perennial grasses	HG lan	specific ↓	insensitive	-	-	-
Hemicryptophytes	H	specific ↓	insensitive	-	-	-
Perennial forbs	HF	insensitive	specific ↑	-	-	-

a PFTs with a sensitive response to grazing had a significant contribution of the grazing-related PC to their final linear models, but other predictor variables had larger effect sizes. PFTs with a specific response to grazing also had a sensitive response, but responded stronger to grazing than to other predictor variables (largest effect size for the grazing-related PC). Insensitive PFTs had a non-significant contribution of grazing-related PC to their final linear models. Arrows indicate the direction of response (↑ positive response to increased grazing pressure, ↓negative response). For details of final linear models refer to Tables S2 and S3.

The direction of response across biomes showed distinct patterns. PFTs that decreased with increasing grazing pressure in the grassland (hemicryptophytes and perennial grasses) responded more strongly to local differences in soil texture and/or bush encroachment in the savanna (Figure 3). PFTs which increased in the grassland decreased in the savanna (annual and small-leaved perennial grasses). Hence, an opposite direction of response was only found in the combination ‘increase in the grassland and decrease in the savanna’.

#### Response consistency across biomes – aggregation level

In the grassland, specific indicators were derived from various degrees of trait aggregation, with three-trait PFTs (small-leaved and broad-leaved perennial grasses) as the most sensitive ones. In contrast, single-trait PFTs (life forms) were rarely sensitive to grazing. Only therophytes were sensitive, but unspecific indicators across biomes. PFTs which were consistent in their specificity (annual grasses; TG) or in their direction of response (perennial grasses; HG) were two-trait aggregations (Table 3). In the savanna, grazing explained the highest proportion of variance for three two-trait PFTs (HG, TG and HF).

### Assessing Land Tenure Effects (Question 3)

**Figure 4:**
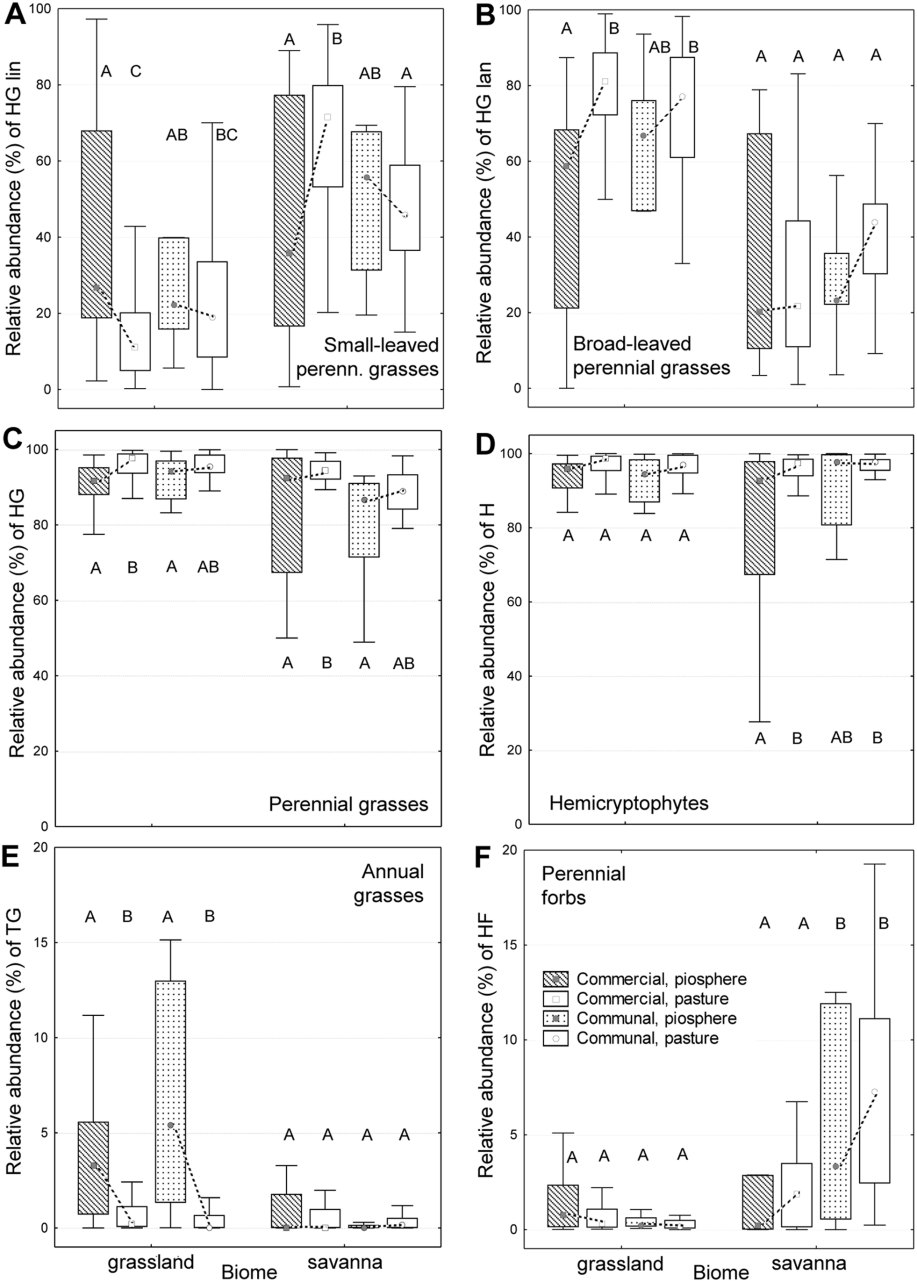
Differences in grazing pressure according to good trait-based indicators (PFTs). Panels A–F compare piosphere and pasture plots across tenure systems (commercial and communal) and biomes (savanna and grassland). All PFTs had a specific response to grazing at least in one biome (see Figure 3 and Table 3). Broken lines connect piosphere and pasture plots of a tenure system within a biome, and different letters indicate significant differences (Tukey’s HSD; *p*<0.05). Boxes show medians and 25^th^ to 75^th^ percentiles, whiskers stand for the non-outlier ranges of the data. Note the different scaling of the y-axis for panels E and F. HG lin = narrow-leaved perennial grasses, HG lan = broad-leaved perennial grasses, HG = perennial grasses, H = hemicryptophytes, TG = annual grasses, HF = perennial forbs.

We used the six PFTs identified as good grazing indicators (Table 3) to evaluate differences in vegetation condition between pasture and piosphere plots in the two tenure systems. We found that tenure-related differences among piosphere and pasture plots were small in both biomes (Figure 4).

Significant effects of tenure system were only detected in the savanna, where mean relative abundances of perennial forbs (HF) on pasture plots indicated a higher grazing pressure on communal farms. However, the response of HF to grazing was rather weak compared to that of the two other specific indicators in this biome (annual and perennial grasses; see Figure 3). One PFT with a sensitive but unspecific response to grazing (HG lin) also indicated tenure-related differences in the savanna.

Significant second- and third-order interactions among biome, tenure system and plot type (Table 4) showed that all plant aggregations responded in an idiosyncratic way to the climatic conditions and to rangeland management. The inclusion of ‘farm’ as a random factor in our analyses revealed that grazing pressure greatly differed between those farms which were nested within a certain tenure system. All indicators agreed with respect to this finding. In both biomes, within-tenure variability tended to be higher on commercial farms than on communal land (higher variability in stocking densities; Table 1). This phenomenon was also visible in the response of specific grazing indicators (see Figure 4).

**Table 4:** Results of partially nested ANOVA with the fixed factors ‘biome’ (grassland or savanna), ‘tenure’ (commercial or communal), ‘type’ (piosphere or pasture plots), and the random factor ‘farm’ nested within ‘tenure’ and ‘biome’.

PFT	Biome		Tenure		Type		Farm		Biome×tenure		Biome×type		Tenure×type		Biome×tenure×type	
	F	*p*	F	*p*	F	*p*	F	*p*	F	*p*	F	*p*	F	*p*	F	*p*
HG lin	46.3	**0.000**	10.8	**0.009**	17.5	**0.000**	2.1	**0.035**	0.34	0.575	4.41	**0.037**	1.51	0.221	1.95	0.164
HG lan	29.5	**0.000**	0.0	0.852	23.2	**0.000**	3.7	**0.000**	0.49	0.502	15.9	**0.000**	0.51	0.475	5.22	**0.024**
HG	12.1	**0.007**	10.7	0.010	15.0	**0.000**	2.5	**0.011**	8.07	**0.020**	2.26	0.135	1.33	0.250	4.73	**0.031**
HF	6.8	**0.026**	2.4	0.152	1.1	0.296	5.6	**0.000**	6.37	**0.030**	0.01	0.907	3.98	**0.047**	4.39	**0.038**
TG	6.6	**0.030**	0.0	0.938	26.4	**0.000**	4.3	**0.000**	0.17	0.711	27.6	**0.000**	1.40	0.238	4.71	**0.032**
H	3.21	**0.075**	1.60	0.208	15.3	**0.000**	3.5	**0.000**	1.28	0.259	6.04	**0.015**	4.69	**0.032**	6.33	**0.013**

Only PFTs with a specific response to grazing at least in one biome (significant contribution of grazing-related principal component to final models, and largest effect size) were tested. HG lin = narrow-leaved perennial grasses, HG lan = broad-leaved perennial grasses, HG = perennial grasses, HF = perennial forbs, TG = annual grasses, H = hemicryptophytes. Significant *p*-values (<0.05) are shown in bold.

## Discussion

### Independence of Soil Gradients and Grazing Gradients (Question 1)

In the first step of our three-step approach, we evaluated if gradients of edaphic site conditions and grazing pressure were independent. We found independent variation in the semi-arid grassland, while in the arid savanna grazing pressure and three mineral nutrients (P, K and Zn) tended to co-vary. Our results are contradictory to many studies reporting strong correlation between soil and grazing gradients due to livestock-mediated changes in soil properties (e.g., [18], [31]). A recent study from the grassland biome also found that mineral nutrients were enriched in zones of highest animal activities [57]. However, changes in these studies were most apparent in the topsoil (0–4 to 0–10 cm). In contrast, we analyzed 0–20 cm samples to capture conditions in the main rooting zone of the grass layer [58]. Here, changes are still detectable but less pronounced, because they are initiated from the soil surface. Our results also confirmed that it is principally possible to identify PFTs which primarily respond to first-order effects of grazing (such as tissue removal) and not to second-order effects such as changes in soil nutrients.

### Poor Response Consistency of PFTs across Biomes (Question 2)

#### Response of community composition

With our study design we sought to maximize detectable effects of grazing by concentrating a large proportion of our sampling effort on the small piosphere zones around water points. At the same time, we aimed to minimize the influence of other environmental conditions in each biome by sampling unburnt sites with similar topography, lithology and soil type.

Our approach was successful in the grassland where grazing pressure (grazing-related PC) was the most important source of variation for community composition. The two ordination methods (DCA and NMDS) agreed with respect to this finding. Ordination results were also strikingly similar with respect to the identity and relative importance of the second and third predictors (mineral nutrients followed by silt and P content in the topsoil; see Fig. 3). In the savanna, though, the first ordination axes were not primarily related to changes in grazing pressure (which only came second or third) but mainly to changes in mineral nutrients, followed by changes in soil texture. This implies a high importance of abiotic site conditions. Our results are not unexpected, as edaphic conditions have often been described to overrule grazing effects, particularly in arid environments. This was found in similar studies from arid southern Kalahari [29], [59], and from arid savannas elsewhere [60], [61]. Soil texture and nutrients played – next to grazing pressure – also an important role for species turnover in the grassland, which is supported by earlier studies [62], [63]. In both biomes, local differences in abiotic site conditions may thus confound grazing-related changes in community composition. The categorical predictor of land tenure explained only a relatively small proportion of species turnover (see Figure 3). We can deduce that the grazing-related PCs successfully captured grazing effects on vegetation, which implies that unaccounted factors were of minor importance for recent community composition.

Note that we were not interested in the ecological relevance of ordination axes per se. Instead, we aimed to quantify the importance of environmental variables in predicting changes in community composition, and to compare these results to changes in PFTs’ relative abundances. For this purpose we used the same statistical procedure (LMs with model selection, and a subsequent calculation of effect sizes) and the same predictor variables (PCA-derived composite variables) for ordination-based and trait-based plant aggregations. It is thus for reasons of comparability that our interpretation of DCA and NMDS axes relied on this standardized approach. Our cross-validation with two indirect ordination methods confirmed that derived results on the relative importance of predictors are sound. The desired quantitative comparison could not have been achieved with often applied direct ordination techniques like CCA.

#### Similarity of PFT and community composition response

In the grassland, the response of broad-leaved and small-leaved perennial grasses to environmental conditions was strikingly similar to the main response of community composition (NMDS 1 and DCA 1). Our observation is supported by a study from a mesic South African grassland which indicated that, along the primary axis of trait variation, broad-leaved grasses occupied one extreme, and narrow-leaved the other [64]. In the savanna, though, the results of the two ordination procedures already disagreed with respect to the relative importance of environmental predictor variables. While none of the PFTs resembled DCA 1 response to environmental conditions, NMDS 1 response was roughly similar to that of perennial grasses (HG), but the relative importance of predictor variables differed. Moreover, the PFT with the highest cumulative effect size (HG lin) responded to four out of five PCs. Thus, species responses to plant-available resources and grazing were apparently too complex as to be reflected by single- to three-trait PFTs.

#### Response consistency across biomes – sensitivity, specificity and direction of response

As grazing-related PCs represent a complex vector combining various processes associated with livestock activities, they should characterize the relevant environmental factors that filter grazing-related plant traits in the studied systems [12]. A specific (negative) response to increasing grazing pressure was found for some dominant PFTs, i.e. small-leaved and broad-leaved perennial grasses in the grassland, and perennial grasses in the savanna. We can thus deduce that these trait combinations were strongly filtered by grazing-related disturbances. The increased relative abundance of annual grasses with higher grazing intensity in the grassland underlines that this PFT has profited from the grazing-induced suppression of the two dominant PFTs (broad-leaved and small-leaved perennial grasses). This is consistent with other observations in semi-arid grasslands [65], [66]. Our results are in broad agreement with the findings of a global analysis of responses to grazing [20]. Considering the same climate-grazing category (drylands with a long evolutionary history of grazing), we also found an inconsistent direction of response for annual plants, a neutral (insensitive) or negative response for graminoid growth forms, and a mostly insensitive response for (both annual and perennial) forbs, except for some perennial forbs which responded positively in the savanna.

The small set of PFTs that had a sensitive response to grazing in both biomes (three out of eight PFTs shared among biomes; Table 3) underlines the difficulty to transfer trait responses to other biomes even within the same climate-grazing category as defined by Díaz et al. [20]. In this context, several patterns of response consistency are of interest. First, some trait combinations only occurred in the semi-arid grassland biome. Second, the direction of response across biomes was not arbitrary; PFTs which decreased in the grassland always responded more strongly to local differences in soil texture and/or bush encroachment in the savanna. These patterns might be explained by the convergence model of drought (aridity) and grazing resistance, stating that aridity and grazing are convergent selective forces [67]. The validity of this model for South Africa’s grasslands has been recently confirmed [68]. Our results suggest that the more arid climate in the savanna has acted as the primary filter [69], and filtered out drought-prone trait combinations (broad-leaved and very broad-leaved perennial grasses) in most habitat types. The occurrence of these PFTs is restricted to habitats with a lower aridity such as sites with favourable edaphic conditions.

The convergence model of aridity and grazing [67] could also explain why an opposite response to grazing always implied an increase of relative abundances in the semi-arid grassland and a decrease in the arid savanna. Along a gradient of additive forces of aridity and grazing (i.e. of environmental harshness), grassland plots under low grazing pressure will occupy the side of most favourable environmental conditions captured in this study, and savanna plots under high grazing pressure the most unfavourable ones. PFTs which appear to have an opposite response to grazing in the two biomes thus rather display an unimodal response along this gradient of environmental harshness captured in this study. For example, the comparatively grazing-tolerant PFT of small-leaved perennial grasses replaced the less tolerant broad-leaved perennial grasses in the grassland when grazing pressure increased. In the more arid savanna, though, small-leaved perennial grasses dominated under conditions of little grazing but were replaced themselves by other PFTs such as annual grasses when grazing pressure increased. This response pattern has also been reported from other arid savannas [30], [70].

#### Response consistency across biomes – aggregation level

We expected that an iterative aggregation of traits into PFTs would allow us to identify good indicators with an optimum level of aggregation. These indicators should capture species’ adaptive response to grazing and ideally be consistent across biomes [16]. We found that one-trait PFTs did either not respond at all, or responded weakly and inconsistently across biomes. This confirms that single traits are insufficient for capturing grazing response [19], which seems particularly true for dryland environments, even on high spatial or organizational scales [20].

Adding traits did not consistently increase the indicative value of PFTs for grazing. In the grassland, three-trait PFTs were the best grazing indicators, but in the savanna, an intermediate (two-trait) level characterized specific grazing indicators. An intermediate level of aggregation also characterized the two indicators which either had a consistent direction (HG) or specificity of response (TG) across biomes. If the inconsistency in response to grazing is inevitable due to the above discussed convergence of aridity and grazing effects, the striking lack of generality is unlikely to be overcome by more refined classification approaches.

Coarse aggregations are obviously more viable for up-scaling across biomes. For example, we found that a dichotomy of perennial versus annual grasses was feasible. Perennial grasses are also a good indicator of ecological services [6] because they are closely linked to a reliable provision of forage biomass [71],[72]. However, the aggregation of ‘Graminoid tall C_4_’ proposed by Díaz et al. [20] for ‘drylands with a long evolutionary history of grazing’ might be inefficient if life history is not included: As tropical and subtropical grasslands and savannas are dominated by C_4_ grasses [73], the proposed aggregation level provides no means of further differentiation.

### Few Tenure-Related Differences in Vegetation State (Question 3)

#### Grazing differences between tenure systems

We assumed that, like in other South African regions [52], [63], communal farms would display stronger grazing-related changes in community composition than commercial farms due to usually higher stocking densities and due to detrimental effects of continuous grazing management on rangeland condition [63]. However, trait-based indicators showed that, in both biomes, tenure-related management effects on vegetation were small, if piospheres were compared to piospheres and pastures to pastures. For farms in the grassland biome, these results are in general agreement with two accompanying studies from the same growing season, focusing on vegetation responses in piospheres based on a taxon-free sampling of vegetative traits [38], and on a time series analysis based on high-resolution spectral imagery of pastures [74].

These results could be explained by large differences in grazing pressure across farms: Within-tenure variability in stocking densities could have masked any between-tenure differences in stocking rates and grazing management (continuous versus rotational). Within each biome, differences in stocking densities (and, similarly, in vegetation state) were larger on commercial farms as compared to communal farms. The grazing management of single owners was thus more variable than on communal farms, where land tenure was shared among a group of users. The greater variability among commercial farms might be associated with recent land reform activities in South Africa [75].

We have used biome-specific indicators for grazing pressure that were relatively insensitive for abiotic site conditions. Differences in abiotic site conditions between commercial and communal rangelands should thus have little confounded our results. For the savanna, we have evidence that such differences occurred: Soil texture parameters (sand and clay content) differed significantly between pasture plots across tenure systems (Table 1). Hence tenure-related differences in the relative abundance of fine-leaved perennial grasses (a PFT which responded in this biome more strongly to soil conditions than to grazing; Table 3) could also be explained by differences in abiotic site conditions. More generally, our results support critical voices stating that changes in vegetation characteristics on rangelands under communal land tenure do not necessarily have to be stronger than those on commercial farms [23].

An alternative, but not mutually exclusive explanation for our observation that tenure-related differences were small relates to the fact that rangeland vegetation outside piospheres generally appeared to be in a good state. In the grassland, this was apparent by the dominance of palatable and preferred species like *Themeda triandra* [28]. Likewise, the dominance of palatable perennial grasses such as *Schmidtia pappophoroides* and *Eragrostis lehmanniana* implied a comparatively good state of savanna pastures [29]. We speculate that the 1–2 years with good rainfall prior to our field study might have created a window of opportunity for regeneration, as postulated [76] and reported [77] for arid and semi-arid rangelands. However we could not differentiate if the recovery of perennial grass cover was due to a recruitment wave (as found in a Namibian savanna when soil moisture was experimentally increased; [78]), or merely due to changes in individual fitness and size. Long-term observations of plant populations, combined with manipulative experiments and vegetation models should allow to track the complex responses of vegetation to grazing and variable climate conditions, and to better understand ecosystem dynamics [79].

## Conclusions

The main aim of this study was to identify functional plant aggregations with a consistent response to grazing across two South African biomes, mainly differing in climatic aridity. The systematic evaluation of hierarchical levels of aggregation was unsuccessful, although we were able to detect a biome-specific optimum level: Three-trait PFTs were the best grazing indicators in the semi-arid grassland, while in the arid savanna two-trait PFTs were better. The striking lack of generalizable PFT responses to grazing might be due to a convergence of aridity and grazing effects, which is unlikely to be overcome by more refined classification approaches. Our study thus presents and advocates for localized indicator sets, as recommended by Díaz et al. [20]. While such sets are useful for applied studies, they do not offer a generic understanding of the combined effects of plant-available resources and grazing on community performance, which would be a prerequisite for an up-scaling of plant responses [80]. For this purpose, a promising approach is to use traits that capture the trade-off between resource acquisition and conservation [2], [51]. Our study also confirms the principal importance of life history (annual versus perennial), growth form, and leaf size in explaining species’ responses to grazing, and advocates for hierarchical combinations of these traits (see [20], [81]). Another important outcome of our research was that certain combinations of traits could successfully detect management effects against the background of a high environmental variability typical for drylands. We think that our novel methodological approach to quantify PFTs’ specificity and sensitivity to grazing pressure, which combines a hierarchical definition of trait-based PFTs with multivariate statistics and model selection procedures, will also be successful for identifying and applying ecological indicators in other ecosystems, and for other environmental drivers.

## Supporting Information

Table S1 **PCA of environmental variables for the grassland and savanna biome.**
(DOC)

Table S2 **Final linear models for the grassland biome, fitted to plant aggregations.**
(DOC)

Table S3 **Final linear models for the savanna biome, fitted to plant aggregations.**
(DOC)

Table S4 **Species list with trait data for the grassland and savanna biome.**
(DOC)

Text S1 **Details on land use history, sampling design and ordinations.**
(DOCX)
