# Supplementary material for: Are There Consistent Grazing Indicators in Drylands? Testing Plant Functional Types of Various Complexity in South Africa’s Grassland and Savanna Biomes
Source: PLoS One. 2014 Aug 11;9(8):e104672. doi: 10.1371/journal.pone.0104672 (PMC4128714; doi:10.1371/journal.pone.0104672)
Supplement: Table S1 — PCA of environmental variables for the grassland and savanna biome. (DOC) [file pone.0104672.s001.doc]

**Table S1** PCA of environmental variables for the grassland and the savanna biome.

| **PCA** | **Grassland biome (n = 121)** | | | | | **Savanna biome (n = 92)** | | | | | | | | |
| --- | --- | --- | --- | --- | --- | --- | --- | --- | --- | --- | --- | --- | --- | --- |
|  | **PC 1** | **PC 2** | **PC 3** | **PC 4** | **PC 5** | **PC 1** | | **PC 2** | | **PC 3** | | **PC 4** | | **PC 5** |
| Axis interpretation | Clay & CEC | Mineral nutrients | Grazing | SOM & Skeleton | Silt & P | Bushes & SOM | | Grazing | | Mineral nutrients | | Silt & Fe | | Clay |
| Variables reflecting grazing pressure | | | | | | | | | | | | | | |
| Distance | 0.012 | 0.255 | **-0.426** | -0.253 | -0.392 | 0.316 | **-0.440** | | -0.252 | | 0.373 | | 0.062 | |
| Pressure | 0.138 | -0.026 | **0.763** | 0.025 | -0.246 | 0.220 | **0.820** | | -0.049 | | -0.014 | | 0.135 | |
| Bare | 0.162 | -0.243 | **0.508** | **-0.562** | 0.076 | 0.067 | **0.882** | | -0.032 | | -0.077 | | -0.114 | |
| Litter | -0.146 | -0.026 | **-0.428** | 0.188 | -0.006 | -0.133 | **-0.560** | | 0.114 | | -0.005 | | 0.292 | |
| Moribund | -0.064 | -0.005 | **-0.800** | -0.077 | -0.168 | -0.270 | **-0.590** | | -0.017 | | **-0.434** | | 0.043 | |
| Bushes | n.a. | n.a. | n.a. | n.a. | n.a. | **0.453** | -0.363 | | 0.183 | | 0.352 | | -0.249 | |
| Soil variables | | | | | | | | | | | | | | |
| Skeleton | 0.220 | 0.061 | 0.163 | **-0.663** | 0.199 | 0.372 | **0.424** | | 0.245 | | -0.326 | | 0.085 | |
| Sand | **-0.776** | -0.095 | 0.028 | -0.240 | -0.398 | -0.233 | -0.025 | | -0.039 | | **-0.410** | | **-0.732** | |
| Silt | 0.086 | -0.001 | -0.050 | -0.050 | **0.819** | 0.126 | -0.015 | | -0.012 | | **0.818** | | 0.129 | |
| Clay | **0.804** | 0.121 | -0.030 | 0.275 | -0.109 | 0.064 | -0.070 | | -0.169 | | -0.102 | | **0.822** | |
| N_tot_ | 0.291 | 0.245 | 0.139 | **0.798** | 0.097 | **0.719** | 0.264 | | 0.077 | | 0.240 | | 0.075 | |
| P_extract_ | 0.057 | 0.222 | -0.039 | 0.056 | **0.685** | 0.077 | **0.659** | | **0.404** | | 0.040 | | 0.165 | |
| K^+^ | 0.323 | **0.490** | 0.050 | 0.036 | 0.217 | 0.292 | **0.523** | | **0.444** | | -0.066 | | 0.149 | |
| Ca^2+^ | **0.682** | -0.361 | 0.159 | -0.016 | 0.084 | **0.896** | 0.028 | | 0.202 | | 0.033 | | 0.046 | |
| Mg^+^ | **0.873** | -0.017 | 0.134 | -0.068 | -0.115 | **0.417** | 0.096 | | **0.532** | | -0.050 | | 0.353 | |
| Na^+^ | **0.676** | -0.171 | 0.096 | 0.070 | 0.116 | 0.046 | 0.358 | | 0.064 | | 0.011 | | **0.477** | |
| Cu^2+^ | **0.426** | **0.578** | -0.064 | 0.169 | -0.152 | 0.104 | 0.140 | | **0.675** | | 0.064 | | -0.231 | |
| Fe^2+^ | -0.277 | **0.824** | -0.050 | 0.144 | 0.017 | -0.408 | 0.066 | | 0.394 | | **0.457** | | -0.072 | |
| Mn^2+^ | -0.058 | **0.827** | -0.105 | 0.095 | 0.058 | 0.083 | 0.172 | | **-0.574** | | 0.030 | | **0.528** | |
| Zn^2+^ | 0.038 | **0.600** | 0.247 | **0.433** | -0.049 | 0.130 | **0.607** | | **0.548** | | 0.141 | | -0.022 | |
| CEC | **0.882** | -0.101 | 0.093 | 0.176 | 0.010 | **0.401** | -0.058 | | **0.631** | | -0.065 | | -0.236 | |
| PH | **0.680** | -0.286 | 0.092 | -0.222 | 0.246 | **0.827** | 0.212 | | -0.211 | | 0.047 | | 0.213 | |
| C_tot_ | **0.465** | 0.163 | 0.072 | **0.761** | 0.076 | **0.887** | 0.150 | | 0.189 | | 0.017 | | 0.018 | |
| Statistics | | | | | | | | | | | | | | |
| Eigenvalue | 4.512 | 2.855 | 2.084 | 2.567 | 1.530 | 4.080 | 3.905 | | 2.612 | | 1.720 | | 2.040 | |
| Total var. [%] | 0.215 | 0.136 | 0.099 | 0.122 | 0.073 | 0.185 | 0.178 | | 0.119 | | 0.078 | | 0.093 | |

PCA (Principal Component Analysis) was performed separately for each biome. Factor loadings (eigenvector coefficients) > 0.4 are in bold. For each Principal Component (PC), eigenvalues and the proportion of total variance explained (Total var. [%]) are given. For axis interpretation, environmental variables with high factor loadings (> 0.4) were used. SOM = soil organic matter, Distance = distance of plot to water point, pressure = estimated grazing pressure based on physical signs of grazing (coded on an ordinal scale from 1 to 9), Bare = bare soil (area of soil not covered by vegetation, litter or skeleton), Litter = proportion of dead biomass on plot surface, Moribund = proportion of moribund biomass on plot surface (previous year’s standing dead). N.a. = not applicable. For details on soil variables, refer to Materials and Methods.
