# Supplementary material for: Are There Consistent Grazing Indicators in Drylands? Testing Plant Functional Types of Various Complexity in South Africa’s Grassland and Savanna Biomes
Source: PLoS One. 2014 Aug 11;9(8):e104672. doi: 10.1371/journal.pone.0104672 (PMC4128714; doi:10.1371/journal.pone.0104672)
Supplement: Table S2 — Final linear models for the grassland biome, fitted to plant aggregations. (DOC) [file pone.0104672.s002.doc]

**Table S2. Final linear models for the grassland biome, fitted to plant aggregations.**

| **Plant aggregation** | **Predictor** | **Details of final linear model (LM)** | | | | | | **Summary of final LM** | | |
| --- | --- | --- | --- | --- | --- | --- | --- | --- | --- | --- |
|  |  | **PC** | **Slope** | **SE** | **t** | ***p*** |  | **∆AIC** | **AIC** | **L Ratio Chi²** |
| DCA1 | Grazing | 3 | 0.443 | 0.047 | 9.382 | < 0.001 | *** | 2.94 | 188.50 | 74.98 |
|  | Min. nutrients | 2 | 0.187 | 0.052 | 3.635 | < 0.001 | *** |  |  |  |
|  | Silt & P | 5 | 0.112 | 0.050 | 2.245 | 0.027 | * |  |  |  |
| NMDS1 | Grazing | 3 | 0.392 | 0.060 | 6.567 | <0.001 | *** | 0.00 | 218.33 | 55.33 |
|  | Min. nutrients | 2 | 0.217 | 0.065 | 3.342 | 0.001 | ** |  |  |  |
|  | Silt & P | 5 | 0.160 | 0.063 | 2.543 | 0.012 | * |  |  |  |
| NMDS 2 | Clay & CEC | 1 | 0.208 | 0.049 | 4.275 | <0.001 | *** | 0.00 | 146.18 | 29.53 |
|  | Tenure CF |  | 0.198 | 0.059 | 3.383 | 0.001 | ** |  |  |  |
| HG lin | Grazing | 3 | 0.157 | 0.023 | 6.725 | < 0.001 | *** | 0.00 | -8.66 | 48.61 |
|  | Min. nutrients | 2 | 0.074 | 0.027 | 2.794 | 0.006 | ** |  |  |  |
|  | Silt & P | 5 | 0.054 | 0.025 | 2.171 | 0.032 | * |  |  |  |
| HG lan | Grazing | 3 | -0.164 | 0.025 | -6.488 | < 0.001 | *** | 0.00 | 7.30 | 49.21 |
|  | Min. nutrients | 2 | -0.087 | 0.029 | -3.020 | 0.003 | ** |  |  |  |
|  | Silt & P | 5 | -0.081 | 0.027 | -3.008 | 0.003 | ** |  |  |  |
| HG ov | Tenure CU |  | -0.096 | 0.022 | -4.405 | < 0.001 | *** | 0.57 | -142.20 | 43.44 |
|  | Min. nutrients | 2 | 0.044 | 0.014 | 3.205 | 0.002 | ** |  |  |  |
|  | Silt & P | 5 | 0.032 | 0.013 | 2.482 | 0.015 | * |  |  |  |
|  | Grazing | 3 | -0.026 | 0.012 | -2.149 | 0.034 | * |  |  |  |
| HG | Tenure CU |  | -0.059 | 0.019 | -3.063 | 0.003 | ** | 0.00 | -168.43 | 26.46 |
|  | Grazing | 3 | -0.033 | 0.011 | -3.096 | 0.003 | ** |  |  |  |
|  | Min. nutrients | 2 | 0.033 | 0.012 | 2.726 | 0.008 | ** |  |  |  |
| HF | Clay & CEC | 1 | -0.024 | 0.006 | -3.926 | 0.000 | ** | 0.00 | -312.00 | 28.12 |
|  | Min. nutrients | 2 | -0.012 | 0.006 | -1.880 | 0.063 |  |  |  |  |
| TG | Grazing | 3 | 0.047 | 0.009 | 5.255 | < 0.001 | *** | 0.00 | 569.57 | 34.80 |
|  | Silt & P | 5 | 0.033 | 0.010 | 3.428 | 0.001 | ** |  |  |  |
|  | Clay & CEC | 1 | 0.032 | 0.010 | 3.095 | 0.003 | ** |  |  |  |
| TF | Tenure CF |  | -0.014 | 0.006 | -2.439 | 0.016 | * | 0.00 | -418.26 | 10.71 |
| Ch | Clay & CEC | 1 | -0.014 | 0.005 | -2.678 | 0.009 | ** | 2.83 | -318.19 | 6.06 |
| H | Grazing | 3 | -0.033 | 0.009 | -3.732 | < 0.001 | *** | 0.00 | -210.05 | 32.16 |
|  | Tenure CF |  | -0.021 | 0.014 | -1.517 | 0.133 |  |  |  |  |
|  | Silt & P | 5 | -0.017 | 0.009 | -1.858 | 0.066 |  |  |  |  |
|  | Clay & CEC | 1 | -0.017 | 0.010 | -1.751 | 0.083 |  |  |  |  |
| T | Clay & CEC | 1 | 0.034 | 0.010 | 3.303 | 0.001 | ** | 2.15 | -197.59 | 29.49 |
|  | Grazing | 3 | 0.029 | 0.009 | 3.069 | 0.003 | ** |  |  |  |
|  | Silt & P | 5 | 0.018 | 0.010 | 1.769 | 0.080 |  |  |  |  |

Tenure system (CF: commercial farms, CU: communal farms) was used as categorical predictor and PCA-derived variables (PC 1-5; see Table S1) were used as linear predictors. The final LM is the best-ﬁtting model based on a likelihood ratio test of all possible subsets of effects (L Ratio; p<0.01 in all cases). Differences to the model with the smallest Akaike Information Criterion (AIC) are given as ∆AIC. Significance of estimates is given with * = *p*< 0.05, ** = *p*< 0.01, *** = *p*<0.001. DCA 1 = plot scores on first DCA axis. For abbreviations of trait-based plant aggregations (PFTs), refer to Table 2.
