# Supplementary material for: Are There Consistent Grazing Indicators in Drylands? Testing Plant Functional Types of Various Complexity in South Africa’s Grassland and Savanna Biomes
Source: PLoS One. 2014 Aug 11;9(8):e104672. doi: 10.1371/journal.pone.0104672 (PMC4128714; doi:10.1371/journal.pone.0104672)
Supplement: Table S3 — Final linear models for the savanna biome, fitted to plant aggregations. (DOC) [file pone.0104672.s003.doc]

***Table S3.*** *Final linear models for the savanna biome, fitted to plant aggregations.*

| **Plant aggregation** | **Predictor** | **Details of final linear model (LM)** | | | | |  | **Summary of final LM** | | |
| --- | --- | --- | --- | --- | --- | --- | --- | --- | --- | --- |
|  |  | **PC** | **Slope** | **SE** | **t** | ***p*** |  | **∆AIC** | **AIC** | **L Ratio Chi²** |
| DCA 1 | Mineral nutrients | 3 | 0.317 | 0.063 | 5.001 | <0.001 | *** | 0.58 | 88.57 | 47.76 |
|  | Grazing | 2 | 0.175 | 0.054 | 3.234 | 0.002 | ** |  |  |  |
|  | Clay | 5 | -0.134 | 0.050 | -2.666 | 0.009 | ** |  |  |  |
|  | Silt & Fe | 4 | 0.104 | 0.047 | 2.203 | 0.031 | * |  |  |  |
| NMDS 1 | Mineral nutrients | 3 | 0.185 | 0.074 | 2.508 | 0.013 | * | 0.00 | 94.95 | 17.20 |
|  | Grazing | 2 | 0.151 | 0.063 | 2.372 | 0.020 | * |  |  |  |
|  | Clay | 5 | -0.126 | 0.055 | -2.311 | 0.024 | * |  |  |  |
| NMDS 2 | Grazing | 2 | -0.517 | 0.048 | -10.26 | <0.001 |  | 0.99 | 60.00 | 90.13 |
|  | Mineral nutrients | 3 | -0.202 | 0.056 | -3.45 | <0.001 |  |  |  |  |
|  | Bushes % -SOM | 1 | -0.201 | 0.065 | -2.94 | 0.002 |  |  |  |  |
|  | Silt % Fe | 4 | -0.111 | 0.043 | -2.44 | 0.011 |  |  |  |  |
| HG lin | Grazing | 2 | -0.134 | 0.032 | -4.142 | <0.001 | *** | 0.00 | 78.94 | 38.38 |
|  | Silt & Fe | 4 | -0.122 | 0.028 | -4.346 | <0.001 | *** |  |  |  |
|  | Bushes & SOM | 1 | -0.114 | 0.043 | -2.664 | 0.009 | ** |  |  |  |
|  | Clay | 5 | -0.063 | 0.030 | -2.105 | 0.039 | * |  |  |  |
| HG lan | Bushes & SOM | 1 | 0.126 | 0.047 | 2.710 | 0.008 | ** | 0.00 | -0.79 | 14.72 |
|  | Silt & Fe | 4 | 0.096 | 0.032 | 2.996 | 0.004 | ** |  |  |  |
|  | Clay | 5 | 0.096 | 0.036 | 2.657 | 0.010 | * |  |  |  |
| HG | Grazing | 2 | -0.188 | 0.034 | -5.609 | <0.001 | *** | 0.00 | 3.41 | 44.87 |
|  | Mineral nutrients | 3 | -0.095 | 0.039 | -2.435 | 0.017 | * |  |  |  |
|  | Silt & Fe | 4 | -0.053 | 0.029 | -1.808 | 0.074 |  |  |  |  |
| HF | Grazing | 2 | -0.064 | 0.032 | -1.980 | 0.051 |  | 1.30 | -69.62 | 28.94 |
|  | Tenure CF |  | 0.049 | 0.019 | 2.656 | 0.009 | ** |  |  |  |
|  | Bushes & SOM | 1 | -0.057 | 0.029 | -1.979 | 0.051 |  |  |  |  |
| TG | Grazing | 2 | -0.033 | 0.012 | -2.813 | 0.006 | ** | 0.00 | -193.62 | 13.98 |
|  | Bushes & SOM | 1 | -0.026 | 0.014 | -1.890 | 0.063 |  |  |  |  |
| TF | Silt & Fe | 4 | 0.026 | 0.008 | 3.154 | 0.002 | ** | 0.00 | -214.87 | 15.30 |
|  | Mineral nutrients | 3 | 0.007 | 0.014 | 0.538 | 0.593 |  |  |  |  |
| H | Silt & Fe | 4 | -0.032 | 0.015 | -2.137 | 0.036 | * | 0.81 | -119.18 | 15.22 |
|  | Mineral nutrients | 3 | -0.026 | 0.024 | -1.056 | 0.295 |  |  |  |  |
|  | Clay | 5 | 0.016 | 0.017 | 0.919 | 0.361 |  |  |  |  |
| T | Silt & Fe | 4 | 0.034 | 0.010 | 3.251 | 0.002 | ** | 1.17 | 348.18 | 21.40 |
|  | Grazing | 2 | -0.029 | 0.013 | -2.226 | 0.029 | * |  |  |  |
|  | Bushes & SOM | 1 | -0.016 | 0.015 | -1.082 | 0.283 |  |  |  |  |

Tenure system (CF: commercial farms, CU: communal farms) was used as categorical predictor and PCA-derived variables (PC 1-5; see Table S1) were used as linear predictors. The final LM is the best-ﬁtting model based on a likelihood ratio test of all possible subsets of effects (L Ratio; p<0.01 in all cases). Differences to the model with the smallest Akaike Information Criterion (AIC) are given as ∆AIC. Significance of estimates is given with * = *p*< 0.05, ** = *p*< 0.01, *** = *p*<0.001. DCA1 = plot scores on first DCA axis, NMDS1, NMDS2 = plots scores on first NMDS axis (second, respectively).. For abbreviations of trait-based plant aggregations (PFTs), refer to Table 2.
