# Supplementary material for: Are There Consistent Grazing Indicators in Drylands? Testing Plant Functional Types of Various Complexity in South Africa’s Grassland and Savanna Biomes
Source: PLoS One. 2014 Aug 11;9(8):e104672. doi: 10.1371/journal.pone.0104672 (PMC4128714; doi:10.1371/journal.pone.0104672)
Supplement: Table S4 — Species list with trait data for the grassland and savanna biome. (DOC) [file pone.0104672.s004.doc]

**Table S4** Species list with trait data for the grassland and savanna biome

| **Species name** | **Authors** | **Family** | **PFT** | **G** | **S** |
| --- | --- | --- | --- | --- | --- |
| Blepharis diversispina | (Nees) C.B.Clarke | Acanthaceae | Ch | X |  |
| Chamaephyte sp.1 |  |  | Ch | X |  |
| Chamaephyte sp.2 |  |  | Ch | X |  |
| Chamaephyte sp.3 |  |  | Ch |  | X |
| Chrysocoma oblongifolia | DC. | Asteraceae | Ch | X |  |
| Crotalaria orientalis ssp. orientalis | Verd. | Fabaceae | Ch |  | X |
| Elephantorrhiza elephantina | (Burch.) Skeels | Fabaceae | Ch |  | X |
| Euphorbia clavarioides | Boiss. | Euphorbiaceae | Ch | X |  |
| Felicia filifolia | (Vent.) Burtt Davy | Asteraceae | Ch | X |  |
| Gnaphalium declinatum | L.f. | Asteraceae | Ch | X |  |
| Gnidia polycephala | Gilg ex Engl. | Asteraceae | Ch |  | X |
| Helichrysum cf. asperum | (Thunb.) Hilliard & B.L.Burtt | Asteraceae | Ch | X |  |
| Hermannia multiflora | Xcq. | Sterculiaceae | Ch | X |  |
| Hermannia tomentosa | (Turcz.) Schinz ex Engl. | Sterculiaceae | Ch | X | X |
| Lycium spec. |  | Solanaceae | Ch | X |  |
| Nolletia arenosa | O.Hoffm. | Asteraceae | Ch |  | X |
| Pentzia incana | (Thunb.) Kuntze | Asteraceae | Ch | X |  |
| Phyllanthus maderaspatensis | L. | Euphorbiaceae | Ch | X |  |
| Rhynchosia totta | (Thunb.) DC. | Fabaceae | Ch | X |  |
| Salvia repens | Burch. ex Benth. | Fabaceae | Ch | X |  |
| Walafrida densiflora | Rolfe | Scrophulariaceae | Ch | X |  |
| Androcymbium cf. melanthioides | Willd. | Colchicaceae | GeF | X |  |
| Geophyte sp.1 |  | Liliaceae | GeF | X |  |
| Geophyte sp.2 |  | Liliaceae | GeF | X |  |
| Geophyte sp.3 |  | Liliaceae | GeF | X |  |
| Geophyte sp.4 |  | Liliaceae | GeF | X |  |
| Geophyte sp.5 |  | Liliaceae | GeF | X |  |
| Geophyte sp.6 |  | Liliaceae | GeF | X |  |
| Geophyte sp.7 |  | Liliaceae | GeF |  | X |
| Iridaceae spec. |  | Iridaceae | GeF | X |  |
| Ophioglossum vulgatum | L. | Ophioglossaceae | GeF | X |  |
| Cyperaceae spec. |  | Cyperaceae | GeG | X |  |
| Cyperus rupestris | Kunth | Cyperaceae | GeG | X |  |
| Cyperus usitatus | Burch. ex Roem. & Schult. | Cyperaceae | GeG | X |  |
| Aizoaceae spec. |  | Aizoaceae | HF | X |  |
| Alternanthera sessilis | (L.) R.Br. ex DC. | Amaranthaceae | HF | X |  |
| Amaranthaceae spec. |  | Amaranthaceae | HF |  | X |
| Aptosimum lineare | Marloth & Engl. | Scrophulariaceae | HF |  | X |
| Aptosimum procumbens | (Lehm.) Burch. ex Steud. | Scrophulariaceae | HF | X |  |
| Arctotis arctotoides | (L.f.) O.Hoffm. | Asteraceae | HF | X |  |
| Argyrolobium lotoides | Trautv. | Fabaceae | HF | X |  |
| Asparagus africanus | Lam. | Asparagaceae | HF |  | X |
| Asteraceae sp.1 |  | Asteraceae | HF | X |  |
| Asteraceae sp.2 |  | Asteraceae | HF | X |  |
| Asteraceae sp.3 |  | Asteraceae | HF | X |  |
| Asteraceae sp.4 |  | Asteraceae | HF | X |  |
| Asteraceae sp.5 |  | Asteraceae | HF |  | X |
| Asteraceae sp.6 |  | Asteraceae | HF | X |  |
| Asteraceae sp.7 |  | Asteraceae | HF | X |  |
| Blepharis integrifolia | (L.f.) E.Mey. & Drège ex Schinz | Acanthaceae | HF | X |  |
| Commelina africana | L. | Acanthaceae | HF | X |  |
| Crassulaceae spec. |  | Crassulaceae | HF | X |  |
| Craterocapsa spec. |  | Campanulaceae | HF | X |  |
| Cyphia triphylla | E.Phillips | Campanulaceae | HF | X |  |
| Didelta spec. |  | Asteraceae | HF | X |  |
| Fimbristylis triflora | (L.) K.Schum. ex Engl. | Cyperaceae | HF | X |  |
| Geraniaceae spec. |  | Geraniaceae | HF | X |  |
| Helichrysum luteoalbum | (L.) Rchb. | Asteraceae | HF | X |  |
| Herbaceous sp.1 |  |  | HF |  | X |
| Herbaceous sp.2 |  |  | HF | X |  |
| Herbaceous sp.3 |  |  | HF |  | X |
| Hermannia depressa | N.E. Br. | Sterculiaceae | HF | X |  |
| Hermannia sp.1 |  | Sterculiaceae | HF | X |  |
| Hibiscus pusillus | Thunb. | Malvaceae | HF | X |  |
| Hibiscus trionum | L. | Malvaceae | HF | X |  |
| Hoffmannseggia burchellii ssp. burchellii | (DC.) Oliv. | Fabaceae | HF |  | X |
| Indigofera sp.2 |  | Fabaceae | HF | X |  |
| Ipomoea spec. |  | Convolvulaceae | HF | X |  |
| Xsione spec. |  | Asteraceae | HF | X |  |
| Kohautia cynanchica | DC. | Rubiaceae | HF |  | X |
| Lessertia prostrata | DC. | Fabaceae | HF | X |  |
| Lessertia sp.1 |  | Fabaceae | HF |  | X |
| Lessertia sp.2 |  | Fabaceae | HF | X |  |
| Macroptilium spec. |  | Fabacaee | HF | X |  |
| Malvaceae spec. |  | Malvaceae | HF | X |  |
| Medicago laciniata | (L.) Mill. | Fabaceae | HF | X |  |
| Merremia verecunda | Rendle | Convolvulaceae | HF |  | X |
| Nenax microphylla | (Sond.) Salter | Rubiaceae | HF | X |  |
| Perennial forbs |  |  | HF |  | X |
| Polygala hottentotta | C. Presl. | Polygalaceae | HF | X |  |
| Pseudognaphalium undulatum | (L.) Hilliard & B.L.Burtt. | Asteraceae | HF | X |  |
| Ranunculus multifidus | Forssk. | Ranunculaceae | HF | X |  |
| Rumex spec. |  |  | HF | X |  |
| Senecio cf. burchellii | DC. | Asteraceae | HF | X |  |
| Senecio cf. engleranus | O.Hoffm. | Asteraceae | HF | X |  |
| Senecio cf. matricariifolius | DC. | Asteraceae | HF | X |  |
| Solanum panduriforme | E. Mey. | Solanaceae | HF | X |  |
| Sonchus dregeanus |  | Asteraceae | HF | X |  |
| Trifolium africanum | Ser. | Fabaceae | HF | X |  |
| Wahlenbergia undulata | (L.f.) A.DC. | Campanulaceae | HF |  | X |
| Andropogon schirensis | Hochst. | Poaceae | HG lan | X |  |
| Anthephora pubescens | Nees | Poaceae | HG lan |  | X |
| Aristida congesta ssp. congesta | Roem. & Schult. | Poaceae | HG lan | X | X |
| Aristida meridionalis | Henrard | Poaceae | HG lan |  | X |
| Cyperaceae sp.5 |  | Cyperaceae | HG lan | X |  |
| Digitaria milanjiana | (Rendle) Stapf. | Poaceae | HG lan | X |  |
| Digitaria spec. |  | Poaceae | HG lan |  | X |
| Eragrostis echinochloidea | Stapf | Poaceae | HG lan | X |  |
| Eragrostis pallens | Hack. | Poaceae | HG lan |  | X |
| Eragrostis plana | Nees | Poaceae | HG lan | X |  |
| Eragrostis superba | Peyr. | Poaceae | HG lan | X |  |
| Heteropogon contortus | (L.) P.Beauv. ex Roem. & Schult. | Poaceae | HG lan | X |  |
| Kyllinga cf erecta | Schumach. | Cyperaceae | HG lan | X |  |
| Panicum kalaharense | Mez | Poaceae | HG lan |  | X |
| Paspalum dilatatum | Poir. | Poaceae | HG lan | X |  |
| Poaceae sp.1 |  | Poaceae | HG lan | X |  |
| Poaceae sp.2 |  | Poaceae | HG lan |  | X |
| Pogonarthria squarrosa | (Roem. & Schult.) Pilg. | Poaceae | HG lan | X |  |
| Sacciolepis cf. africana | C.E.Hubb. & Snowden | Poaceae | HG lan | X |  |
| Schmidtia pappophoroides | Steud. ex J.A.Schmidt | Poaceae | HG lan |  | X |
| Setaria incrassata | (Hochst.) Hack. | Poaceae | HG lan | X |  |
| Sporobolus ioclados | (Trin.) Nees | Poaceae | HG lan | X | X |
| Themeda triandra | Forssk. | Poaceae | HG lan | X |  |
| Trichoneura grandiglumis | (Nees) Ekman | Poaceae | HG lan | X |  |
| Triraphis andropogonoides | (Steud.) E.Phillips | Poaceae | HG lan | X |  |
| Aristida diffusa ssp. diffusa | Trin. | Poaceae | HG lin | X |  |
| Aristida stipitata ssp. stipitata | Hack. | Poaceae | HG lin |  | X |
| Aristida vestita | Thunb. | Poaceae | HG lin | X | X |
| Cymbopogon pospischilii | (K.Schum.) C.E.Hubb. | Poaceae | HG lin | X |  |
| Cynodon cf. |  | Poaceae | HG lin | X |  |
| Cynodon dactylon | (L.) Pers. | Poaceae | HG lin | X |  |
| Cynodon hirsutus | Stent | Poaceae | HG lin | X | X |
| Eragrostis capensis | (Thunb.) Trin. | Poaceae | HG lin | X |  |
| Eragrostis curvula | (Schrad.) Nees | Poaceae | HG lin | X |  |
| Eragrostis cylindriflora | Hochst. | Poaceae | HG lin |  | X |
| Eragrostis lehmanniana | Nees | Poaceae | HG lin |  | X |
| Eragrostis obtusa | Munro ex Ficalho & Hiern | Poaceae | HG lin | X |  |
| Helictotrichon imberbe | (Nees) Veldkamp | Poaceae | HG lin | X |  |
| Microchloa caffra | Nees | Poaceae | HG lin | X |  |
| Pennisetum macrourum | Trin. | Poaceae | HG lin | X |  |
| Pennisetum sphacelatum | (Nees) T.Durand & Schinz | Poaceae | HG lin | X |  |
| Setaria sphacelata var. sphacelata | (Stapf) Clayton | Poaceae | HG lin | X |  |
| Sporobolus fimbriatus | (Trin.) Nees | Poaceae | HG lin | X |  |
| Stipagrostis uniplumis var. uniplumis | (Licht.) De Winter | Poaceae | HG lin | X | X |
| Tragus koelerioides | Asch. | Poaceae | HG lin | X | X |
| Digitaria eriantha | Steud. | Poaceae | HG ov | X |  |
| Panicum coloratum | L. | Poaceae | HG ov | X |  |
| Alternanthera pungens | Kunth | Amaranthaceae | TF | X |  |
| Amaranthaceae sp.2 |  | Amaranthaceae | TF | X |  |
| Amaranthus hybridus | L. | Amaranthaceae | TF |  | X |
| Bidens biternata | (Lour.) Merr. & Scherff | Asteraceae | TF | X |  |
| Boerhavia repens | L. | Nyctaginaceae | TF |  | X |
| Chenopodium album | L. | Chenopodiaceae | TF | X | X |
| Commelina benghalensis | L. | Commelinaceae | TF | X |  |
| Conyza bonariensis | (L.) Cronquist | Asteraceae | TF | X |  |
| Crotalaria distans | Benth. | Fabaceae | TF | X |  |
| Euphorbia inaequilatera var. inaequilatera | Sond. | Euphorbiaceae | TF | X |  |
| Annual forbs |  |  | TF |  | X |
| Galenia africana | L. | Aizoaceae | TF |  | X |
| Geigeria spec. |  | Asteraceae | TF | X |  |
| Helichrysum argyrosphaerum | DC. | Asteraceae | TF |  | X |
| Heliotropium ciliatum | Kaplan | Boraginaceae | TF |  | X |
| Herbaceous sp.1 |  |  | TF | X |  |
| Herbaceous sp.2 |  |  | TF | X |  |
| Herbaceous sp.3 |  |  | TF | X |  |
| Herbaceous sp.4 |  |  | TF | X |  |
| Hermbstaedtia fleckii | Bak. & C. B. Cl. | Amaranthaceae | TF |  | X |
| Hibiscus spec. |  | Malvaceae | TF | X |  |
| Indigofera sp.1 |  | Fabaceae | TF | X |  |
| Indigofera sp.2 |  | Fabaceae | TF |  | X |
| Jamesbrittenia aurantiaca | (Burch.) Hilliard | Asteraceae | TF | X |  |
| Lamiaceae spec. |  | Lamiaceae | TF | X |  |
| Lepidium africanum | (Burm.f.) DC. | Brassicaceae | TF | X |  |
| Limeum myosotis var. myosotis | H.Walter | Molluginaceae | TF |  | X |
| Lotus spec. |  | Fabaceae | TF | X |  |
| Monsonia senegalensis | Guill. & Perr. | Geraniaceae | TF | X |  |
| Nidorella resedifolia | DC. | Asteraceae | TF | X |  |
| Oxalis sp.1 |  | Oxalidaceae | TF | X |  |
| Oxalis sp.2 |  | Oxalidaceae | TF |  | X |
| Oxalis sp.3 |  | Oxalidaceae | TF | X |  |
| Phyllanthus angolensis | Müll.Arg. | Euphorbiaceae | TF |  | X |
| Poaceae spec. |  | Poaceae | TF |  | X |
| Rubiaceae spec. |  | Rubiaceae | TF | X |  |
| Senecio sp.1 |  | Asteraceae | TF |  | X |
| Senecio sp.2 |  | Asteraceae | TF | X |  |
| Senna italica | Mill. | Fabaceae | TF |  | X |
| Sesamum triphyllum | Welw. ex Asch. | Pedaliaceae | TF |  | X |
| Tagetes minuta | L. | Asteraceae | TF | X |  |
| Tephrosia spec. |  | Fabaceae | TF | X |  |
| Tribulus terrestris | L. | Zygophyllaceae | TF | X | X |
| Urochloa panicoides | P.Beauv. | Poaceae | TF | X |  |
| Wahlenbergia spec. |  | Campanulaceae | TF | X |  |
| Brachiaria eruciformis | (Sm.) Griseb. | Poaceae | TG lan | X |  |
| Brachiaria marlothii | (Hack.) Stent | Poaceae | TG lan |  | X |
| Chloris virgata | Sw. | Poaceae | TG lan | X |  |
| Digitaria ternata | (A.Rich.) Stapf | Poaceae | TG lan | X |  |
| Enneapogon cenchroides | (Roem. & Schult.) C.E.Hubb. | Poaceae | TG lan |  | X |
| Melinis repens ssp. repens | (Willd.) Zizka | Poaceae | TG lan |  | X |
| Schmidtia kalahariensis | Stent | Poaceae | TG lan |  | X |
| Tragus racemosus | (L.) All. | Poaceae | TG lan | X |  |
| Aristida adscensionis | L. | Poaceae | TG lin | X | X |
| Aristida bipartita | (Nees) Trin. & Rupr. | Poaceae | TG lin | X |  |
| Bulbostylis humilis | (Kunth) C.B.Clarke | Cyperaceae | TG lin | X |  |

PFT: Finest PFT classification following the hierarchical, three-level aggregation procedure of traits. Acronyms describe the combination of traits, with the first 1-2 letters for life form (here: Ch = chamaephytes (shrubs), Ge = geophytes, H = hemicryptophytes (perennial forbs and graminoids), T theropyhtes (annuals), followed by an acronym for growth form (G = graminoids, F = forbs), for graminoids, acronyms for leaf width are added (lin = linear (narrow-leaved), < 5 mm; lan = lanceolate (broad-leaved), 5-10 mm, ov = ovate (very broad-leaved), > 10 mm). Species’ occurrence in the two South African study areas is coded with G for grassland biome and S for savanna biome.
