# Supplementary material for: Are There Consistent Grazing Indicators in Drylands? Testing Plant Functional Types of Various Complexity in South Africa’s Grassland and Savanna Biomes
Source: PLoS One. 2014 Aug 11;9(8):e104672. doi: 10.1371/journal.pone.0104672 (PMC4128714; doi:10.1371/journal.pone.0104672)
Supplement: Text S1 — Details on land use history, sampling design and ordinations. (DOCX) [file pone.0104672.s005.docx]

**Text S1 Details on land use history, sampling design and ordinations.**

*Land use history*. In the grassland, commercial farms were allocated to individual owners during the late nineteenth century [[1](#_ENREF_1)], and boreholes were drilled in the following decades. CU rangelands were fenced off into grazing camps in the early 1960s [[2](#_ENREF_2)], and boreholes were drilled then. After the breakdown of apartheid in 1994, most fences were unmaintained and broke down [[1](#_ENREF_1)], and only a small proportion of boreholes was maintained, increasing the grazing pressure around them. In the savanna, all water points were drilled between the 1930ies and the early 1960ies (Louw van der Walt, pers. comm.). All grazing gradients included in this study are thus at least fifty years old.

Stocking densities on CF are reported to be mostly kept around recommended numbers which are ca. 5-10 ha per large stock unit (LSU) for grassland pastures in good condition [[3](#_ENREF_3)], and ca. 9-16 ha LSU^-1^ for the arid savanna at the southern fringes of the Kalahari [[4](#_ENREF_4)]. CU rangelands are generally reported to have higher stocking densities than CF rangelands [[5](#_ENREF_5)].

*Sampling design.* We aimed to minimize potentially confounding effects of other environmental conditions. We specifically addressed (i) climate, (ii) soil, topography and geology, and (iii) fire. For climate, we contrasted two levels of climatic aridity (arid and semi-arid). Collecting data with a unified sampling design in two climatically different regions is superior to most previous studies, and allows more direct inferences as the meta-analyses and reviews available so far. In both biomes, we selected a relatively small study area to minimize differences in rainfall among sites. To reduce differences in geology, soil type or water relations within biomes, we restricted our sampling to areas with the same lithology and soil type (lixisols in the grassland biome, arenosols in the savanna; see Table 1). This was done in close co-operation with researchers from a parallel study on soil degradation [[for the grassland biome see 1](#_ENREF_1)]. Moreover, we limited our sampling to plains and only sampled around artificial water points (boreholes; except one dam on a commercial farm in the grassland biome) to reduce potentially confounding effects of topography-related differences in abiotic site conditions. Small-scale variation in soil physical and chemical properties was addressed by collecting detailed soil information. In both regions, fire is generally not a management tool used by farmers, and wildfires are usually excluded [[6](#_ENREF_6)]. Personal observations and interviews with farmers confirmed that fire disturbance did not occur on sampled pastures, neither in the sampling year nor in the years prior to our investigation. Due to our research objective to compare tenure-related differences in grazing pressure, we could not control for stocking density by selecting only farms with similar stocking densities. To take account for the variation of grazing impact between farms and along local grazing gradients, we used several proxies to estimate grazing pressure on the plot level [[7](#_ENREF_7)].

*Details on DCA.* Preliminary DCA showed that the underlying gradient along the first axis was in both biomes between 3 and 4 SD (grassland: 3.7 SD; eigenvalue 0.41; savanna: 3.9 SD; eigenvalue 0.61), linear and unimodal models were principally suitable [[48](#_ENREF_48)]; we have chosen unimodal models to aggregate within-biome species turnover into scores of the first DCA axis (DCA 1).

*Details on NMDS.* Initial ordinations were performed from random starting points and for 200 iterations, stepping down from six dimensions to a 1-dimensional solution. We compared real-data runs (n = 10) to the null model of randomized data runs (n = 100) via Monte Carlo tests (at *p* < 0.05), and selected a reduced dimensionality via scree plots of NMDS stress values. For both biomes, the greatest reduction in stress for the ordination was achieved with a two-dimensional solution (NMDS 1 and NMDS 2). The best solution had a final stress value of 13.62 for the grassland and 17.38 for the savanna, and a final instability <10^-3^ (0.002, respectively) achieved after 58 (59) iterations, indicating acceptable results for both analyses.

**References**

1. Kotzé E, Sandhage-Hofmann A, Meinel JA, du Preez CC, Amelung W (2013) Rangeland management impacts on the properties of clayey soils along grazing gradients in the semi-arid grassland biome of South Africa. Journal of Arid Environments 97: 220-229.

2. Naumann C (2014) Stability and transformation in a South African landscape: rural livelihoods, governmental interventions and agro-economic change in Thaba Nchu. Journal of Southern African Studies 40: 41-57.

3. van der Westhuizen HC, Snyman HA, van Rensburg WLJ, Potgieter JHJ (2001) The quantification of grazing capacity from grazing - and production values for forage species in semi-arid grasslands of southern Africa. African Journal of Range & Forage Science 18: 43-52.

4. Perkins JS, Thomas DSG (1993) Spreading deserts or spatially confined environmental impacts? Land degradation and cattle ranching in the Kalahari desert of Botswana. Land Degradation and Rehabilitation 4: 179-194.

5. Vetter S, Goqwana WM, Bond WJ, Trollope WW (2006) Effects of land tenure, geology and topography on vegetation and soils of two grassland types in South Africa. African Journal of Range & Forage Science 23: 13-27.

6. Booysen PdV, Tainton NM (1984) Ecological effects of fire in South African ecosystems. Berlin: Springer Verlag.

7. Pyke DA, Herrick JE, Shaver P, Pellant M (2002) Rangeland health attributes and indicators for qualitative assessment. Journal of Range Management 55: 584-597.
